# A Network Pharmacology-Based Strategy for Unveiling the Mechanisms of Tripterygium Wilfordii Hook F against Diabetic Kidney Disease

**DOI:** 10.1155/2020/2421631

**Published:** 2020-11-20

**Authors:** Yuyang Wang, Tongtong Liu, Fang Ma, Xiaoguang Lu, Huimin Mao, Weie Zhou, Liping Yang, Ping Li, Yongli Zhan

**Affiliations:** ^1^Department of Nephrology, Guang'anmen Hospital of China Academy of Traditional Chinese Medical Sciences, Beijing 100053, China; ^2^Tianjin University of Traditional Chinese Medicine, Tianjin 301617, China; ^3^Beijing Key Laboratory for Immune-Mediated Inflammatory Diseases, Institute of Clinical Medical Sciences, China-Japan Friendship Hospital, Beijing 100029, China

## Abstract

**Background:**

Diabetic kidney disease (DKD) poses a major public-health burden globally. Tripterygium wilfordii Hook F (TwHF) is a widely employed herbal medicine in decreasing albuminuria among diabetic patients. However, a holistic network pharmacology strategy to investigate the active components and therapeutic mechanism underlying DKD is still unavailable.

**Methods:**

We collected TwHF ingredients and their targets by traditional Chinese Medicine databases (TCMSP). Then, we obtained DKD targets from GeneCards and OMIM and collected and analyzed TwHF-DKD common targets using the STRING database. Protein-protein interaction (PPI) network was established by Cytoscape and analyzed by MCODE plugin to get clusters. In addition, the cytoHubba software was used to identify hub genes. Finally, all the targets of clusters were subjected for Gene Ontology (GO) and Kyoto Encyclopedia of Genes and Genomes (KEGG) pathway enrichment analyses via DAVID.

**Results:**

A total of 51 active ingredients in TwHF were identified and hit by 88 potential targets related to DKD. Compounds correspond to more targets include kaempferol, beta-sitosterol, stigmasterol, and Triptoditerpenic acid B, which appeared to be high-potential compounds. Genes with higher degree including VEGFA, PTGS2, JUN, MAPK8, and HSP90AA1 are hub genes of TwHF against DKD, which are involved in inflammation, insulin resistance, and lipid homeostasis. Kaempferol and VEGFA were represented as the uppermost active ingredient and core gene of TwHF in treating DKD, respectively. DAVID results indicated that TwHF may play a role in treating DKD through AGE-RAGE signaling pathway, IL-17 signaling pathway, TNF signaling pathway, insulin resistance, and calcium signaling pathway (*P* < 0.05).

**Conclusion:**

Kaempferol and VEGFA were represented as the uppermost active ingredient and core gene of TwHF in treating DKD, respectively. The key mechanisms of TwHF against DKD might be involved in the reduction of renal inflammation by downregulating VEGFA.

## 1. Introduction

Diabetic kidney disease (DKD), a highly prevalent microvascular complication of diabetes, is characterized by thickening of the glomerular basement membrane, mesangial expansion, podocyte, and glomerular injury, leading to glomerular sclerosis and tubulointerstitial fibrosis [1]. It occurs in almost 20-50% patients with type 1 or type 2 diabetes worldwide, and even in the presence of adequate glycemic control, it remains the main cause of morbidity and mortality among diabetic patients [2, 3]. Since those living with DKD are at higher risks of progression to cardiovascular comorbidities and end-stage kidney disease (ESKD), thus DKD is emerging as a major healthcare challenge around the globe. The clinical hallmark of DKD is described as the presence of progressive decline in renal function and persistent excretion of urinary albumin. Several mechanisms contribute to DKD pathogenesis through driving an overproduction of advanced glycation end products, hemodynamic changes, inflammation, oxidative stress, fibrosis, endothelial, and podocyte injury, ultimately leading to albuminuria and reduced renal function [3]. Current standard therapies to manage DKD recommend drugs controlling blood pressure and hyperglycemia, mainly including renin angiotensin aldosterone system (RAAS) inhibitors, sodium glucose cotransporter 2 (SGLT2) inhibitors, and glucagon-like peptide 1 (GLP1) receptor agonist. Despite those strategies showing promising results in DKD, many diabetic patients continue to progress to DKD and ESKD [3, 4]. The multifactorial origin of DKD and the multiple underlying molecular mechanisms involved make it difficult to achieve successful results with one single drug. Novel therapeutic agents based on multiple targets contribute to DKD progression are therefore urgently required.

Guided by accumulative empirical experience, Chinese herbal medicine [5] acts as an effective therapy in the treatment of DKD for centuries in China and some Asian countries. Different from chemical medications, it is reported that Chinese herbal medicine [5] targets on diverse pathways to restore cellular homeostasis, which may provide a new option for the treatment of multifactorial diseases like DKD [6]. Tripterygium wilfordii Hook F (TwHF) is the most well-known drug for DKD among CHM and exerts a remarkable effect in decreasing albuminuria excretion. Evidence obtained from clinical trials suggested treatment of TwHF in combination of ARB/ACEI is more effective than ARB/ACEI monotherapy in reduction of proteinuria for DKD patients [7, 8]. A wide spectrum of biological activities about TwHF including anti-inflammatory, immunosuppressive, antioxidation, and antifibrosis has been demonstrated in the last decades [9–13], which may attribute to its renoprotective effects against DKD [14, 15]. However, a satisfactory explanation of its pharmacological mechanisms and material bases related to DKD remains unavailable at the moment.

**Figure 1:**
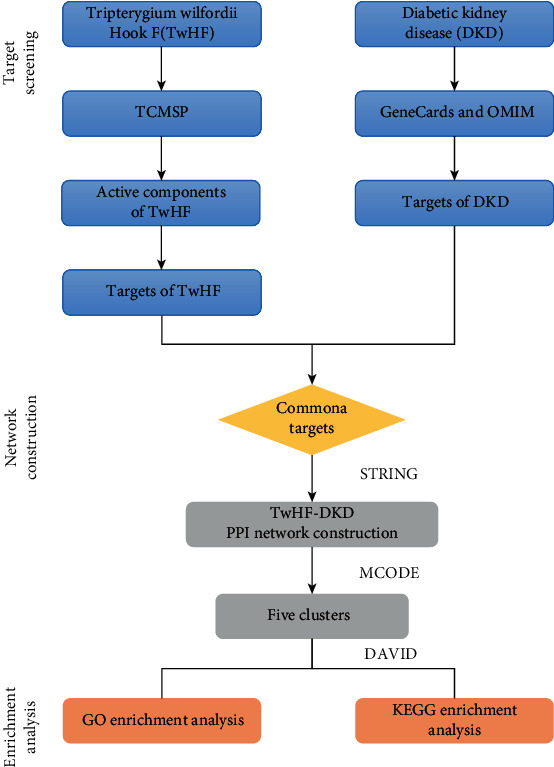
The whole framework for TwHF against DKD.

Network pharmacology is an interactive network used to study “compound-proteins/genes-disease” pathways, which own the ability of elucidating complexities for biological systems, drugs, and diseases from a systemic perspective [16, 17]. This concept shares a quite similar holistic philosophy as CHM, which supplies a comprehensive method to systematically reveal the interactions between multicomponents, multitargets, and multipathways of the active ingredients in CHM [18, 19]. This study is aimed at exploring the pharmacological mechanisms of TwHF for DKD employing network pharmacology approach. Our workflow was exhibited in Figure 1.

## 2. Material and Methods

### 2.1. TwHF Database Construction and Prediction of Target Genes

The Traditional Chinese Medicine Systems Pharmacology Database (TCMSP, http://tcmspw.com/tcmsp.php) was employed to collect the chemical compounds of TwHF, which is a unique systematic pharmacology platform of CHM that displays the associations between drugs, targets, and diseases [20]. We screened the TwHF compounds using ADME (absorption, distribution, metabolism, and excretion) models, and the ingredients with OB (oral bioavailability (OB)) ≥30% and the DL (drug-likeness (DL)) ≥0.18 were screened out as candidate compounds for further analysis according to a suggested criterion by the TCMSP database. Meanwhile, the target genes related to active ingredients of TwHF were gathered based on the TCMSP database. UniProt database (https://www.uniprot.org) provides access to a vast amount of sequence and functional information of proteins in a comprehensive, high-quality, and freely accessible way for scientists worldwide [21]. All obtained targets were subsequently put into UniProtKB, with the organism selected as “Homo sapiens,” to search for official target names. Therefore, we collected the active compound and involved target genes of TwHF, and the details are described in Table S1. Additionally, components correspond to more targets (top 10) were imported into PubChem database (https://pubchem.ncbi.nlm.nih.gov/) for acquiring the compound structures.

### 2.2. Identification of DKD Targets

The keyword “diabetic kidney disease” was used in the GeneCards (https://www.genecards.org) and Online Mendelian Inheritance in Man (OMIM, https://www.omim.org) databases to search for DKD-related targets. GeneCards is a comprehensive, authoritative human gene compendium that enables scientific community to effectively navigate concise genome, transcriptome, proteome, disease, and function data on all predicted and known human genes [22, 23]. OMIM is considered as the primary repository of comprehensive [24], freely accessible information on genes, genetic phenotypes, and gene-phenotype relationships.

### 2.3. Construction and Analysis of the Protein-Protein Interaction (PPI) Network

The intersection of the compound targets and DKD disease targets was determined with the semiprogrammed foot software R project (×64 3.6.1) and taken as the candidate targets for the components of TwHF in the treatment of DKD. A protein-protein interaction (PPI) network of overlapped target genes was built based on the STRING database (http://stringdb.org) with the organism set as “Homo sapiens” [25]. Afterwards, the target interaction information was drawn and analyzed in the visualization software Cytoscape (Version 3.7.2; https://www.cytoscape.org/). The color of the node was displayed in a gradient from red to yellow according to the descending order of the degree value, and a higher degree value node was utilized to present putative crucial targets in the PPI network. Besides, the cytoHubba software was used to select hub genes based on mixed character calculation (MCC) score, and the Molecular Complex Detection (MCODE), a plugin in the Cytoscape software, helped us to screen significant functional modules and get clusters in the PPI network according to K − core = 2.

### 2.4. Functional Enrichment and Pathway Analysis

Gene Ontology (GO) and Kyoto Encyclopedia of Genes and Genomes (KEGG) pathway enrichment analyses for TwHF in DKD were performed using the DAVID (https://david.ncifcrf.gov) database [26]. Results with *P* < 0.05 and *Q* < 0.05 were retained for further analysis about key targets in the PPI network involved in biological processes, molecular functions, cellular components, and functional signaling pathway.

## 3. Results and Discussion

### 3.1. Compound-Compound Target Network

**Figure 2:**
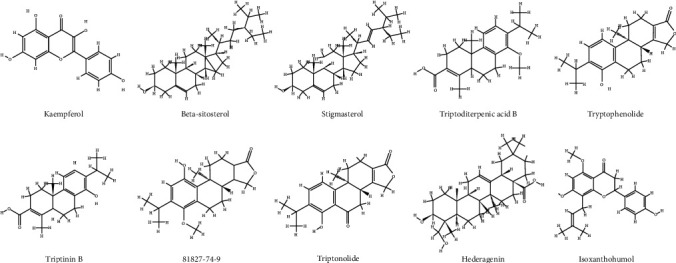
The chemical structure of the ten representative compounds in TwHF.

**Figure 3:**
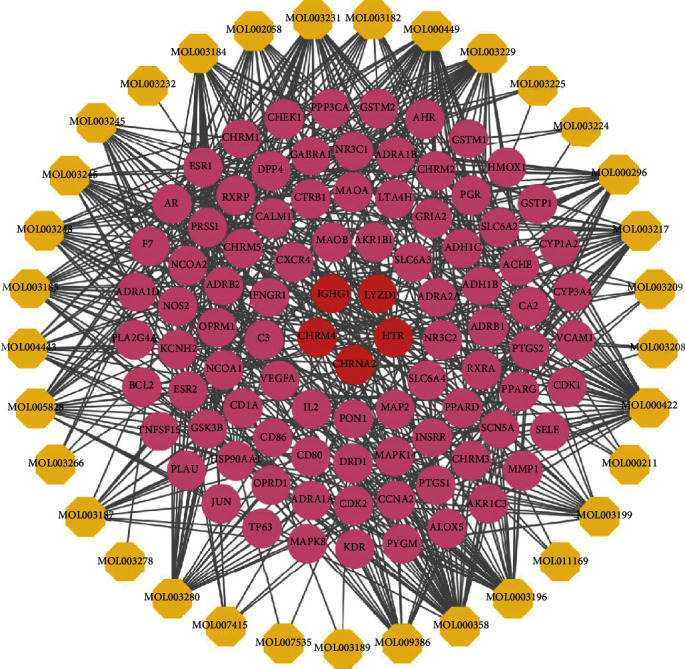
Compound-compound target network of targets in TwHF. The yellow hexagon stands for compounds in TwHF. The nodes represented target genes of TwHF, in which pink nodes stand for common target of TwHF-DKD, and red nodes were employed to represent compound targets not related to DKD. The lines stand for interactions between compounds and target nodes.

The active compound and compound targets were collected via the TCMSP database. There were 51 components in TwHF based on the ADME parameters in TCMSP of OB ≥ 30% and DL ≥ 0.18 and were listed in Table 1 (detailed in Table S1 and Figure 2). Besides, a total of 93 involved targets were obtained after removing the duplicate data. We constructed a compound-compound target network in purpose of further elucidate the interactions between active ingredients of TwHF and corresponding targets, as shown in Figure 3. By mapping 51 compounds linked to 93 targets, the network consists of 126 nodes and 579 edges, in which the components calculated in yellow from TwHF, and the colorized pink circles reflect cotarget genes of TwHF-DKD, and red circles stand for component targets only. The multitarget effect of TwHF was demonstrated in this network.

Compounds correspond to more targets include kaempferol, beta-sitosterol, stigmasterol, and Triptoditerpenic acid B, which associate with 46, 39, 38, and 33 targets, respectively. The result indicated that these compounds might serve as potential ingredients for the treatment of DKD. The antidiabetic activity of kaempferol, beta-sitosterol, and stigmasterol has been reported due to their antioxidant and anti-inflammatory properties while the study on Triptoditerpenic acid B was a void [27–29]. Kaempferol, a natural flavonoid, has been suggested to inhibit hyperglycemia-induced activation of inflammatory cytokines, TGF-*β*1 expression, and oxidative stress in NRK-52E and human renal tubular epithelial cells [29], meanwhile, to prevent lipid accumulation and ER stress in pancreatic *β* cell [30], which indicated the potential of kaempferol for treating DKD. We conducted GO and KEGG analyses of cotargets of Triptoditerpenic acid B and DKD (Table S6), and the results indicated that inflammatory response might act as important mechanisms of Triptoditerpenic acid B in the treatment of DKD.

### 3.2. Compound Target-DKD Target Network Analysis

**Figure 4:**
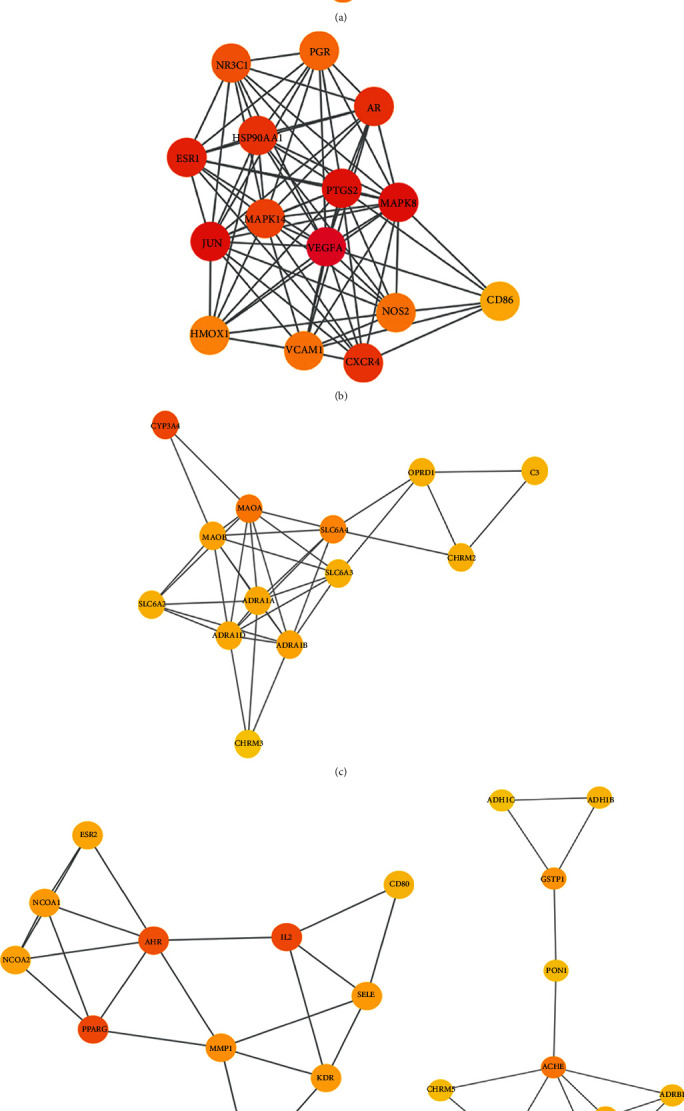
Compound-DKD PPI network and its clusters. (a) TwHF target-DKD target PPI network comprised of 88 nodes and 547 edges. The nodes stand for genes, and the lines stand for the interactions between a pair of target genes; The node color was redder as the degree value increased. (b–f) Clusters of compound-DKD PPI network. Five clusters were obtained in compound-DKD PPI network, and (b–f) stand for clusters 1-5.

Using the GeneCards and OMIM databases, 11389 target genes associated with DKD were retrieved, including 11247 in GeneCards and 142 in OMIM. A total of 11261 targets were obtained after striking out redundant entries. The details are displayed in Table S2. In total, 88 overlapping genes were determined via matching the compound-related 93 target genes with DKD-related 111261 target genes (Figure S1) and were imported into the STRING database where the PPI network was later established for analyzing the relationship between compound targets and disease targets. Almost all of them (88) among a total of 93 targets of TwHF can directly affect DKD-related targets, indicating that TwHF was promising candidates for preventing and treating DKD. As shown in Figure 4(a), this network composed of 88 nodes and 547 edges, and the average node degree is 12.4. The nodes and edges represent target genes and interactions between a pair of target genes, respectively. The color of the node was displayed in a gradient from red to yellow according to the descending order of the degree value.

Targets with higher degrees including VEGFA, PTGS2, JUN, MAPK8, and HSP90AA1 were identified as hub genes of TwHF against DKD based on cytoHubba analysis. These genes are mostly related to chronic inflammation, insulin resistance, lipid homeostasis, oxidative stress, and fibrosis [31–34].

Vascular endothelial growth factor A (VEGFA), a predominant proangiogenic factor in the kidney, is secreted by podocytes and provides essential maintenance signals for endothelial cells, podocytes, and mesangial cells in the glomerulus [35]. Chronic hyperglycemia and excessive production of advanced glycation end products (AGEs) induce VEGFA synthesis and secretion, thereby stimulating abnormalities in multiple signaling pathways including inflammation, excessive ROS generation, TGF-*β* and CTGF activation, and foot process effacement and ultimately leading to extracellular matrix (ECM) accumulation, GBM thickening, and albuminuria [31, 36]. VEGFA inhibitor could lead to remission in albuminuria, glomerular hypertrophy, and endothelial activation in streptozotocin-induced diabetic mice [36]. Prostaglandin synthases (PTGS), including PTGS1 and PTGS2, are known proinflammatory cytokines and have been implicated in the pathogenesis of numerous inflammatory diseases like diabetes [32]. Both enzymes are reported to be found in pancreatic tissue, and there is evidence that PTGS2 is involved in inflammatory mediator-induced damage of pancreatic *β* cells [33]. c-JUN N-terminal kinase (JNK) and p38 mitogen-activated protein kinase (p38 MAPK) are stress-activated protein kinases. Studies have shown that glomerular and tubulointerstitial p38 MAPK/JNK signaling was dramatically activated in renal biopsies from patients diagnosed with diabetes and a number of diabetic animal models [37, 38]. Notably, activation of p38 MAPK has been reported to be strongly associated with renal inflammation and fibrosis in the settings of diabetes, while inhibition of p38 MAPK ameliorated proinflammation and profibrotic responses. In addition, hyperactivation of mitogen-activated protein kinase 8 (MAPK8) plays a pivotal role in the pathogenesis of insulin resistance and obesity, while MAPK8 knockout mice presented improved adiposity and insulin sensitivity [34]. Heat shock protein C (HSP90) is predominantly expressed in podocytes and mesangial cells in the glomeruli. Despite without any changes of HSP90 expression in the renal cortex of type 2 diabetic mice and hyperglycemia incubated glomerular cells [39], HSP90 inhibition has been demonstrated to present a beneficial effect in modulating lipid homeostasis and suppressing renal inflammation as indicated by the inactivation of NF-*κ*B and STAT signaling pathways in DM2 mice [40].

Overall, these results indicated that the underlying mechanism of TwHF in the treatment of DKD may be involved in the reduction of excessive inflammation and protection against islets dysfunction and lipid deposition.

### 3.3. Cluster of Compound-DKD PPI Network

Targets in compound-DKD PPI network were analyzed by MCODE, and 5 central gene clusters were returned (Table 2, Figures 4(b)–4(f)). These clusters were subsequently imported into DAVID for GO enrichment analysis (displayed in Table S3), and numerous DKD-related biological processes were returned including the following: (1) inflammatory and immune response: response to lipopolysaccharide (GO:0032496), regulation of inflammatory response (GO:0050727), regulation of T-helper cell differentiation (GO:0045622), regulation of leukocyte proliferation (GO:0070663), regulation of lymphocyte proliferation (GO:0050670), and regulation of B cell proliferation (GO:0030888); (2) hypoxia and oxidative stress: response to hypoxia (GO:0001666), response to decreased oxygen levels (GO:0036293), reactive oxygen species metabolic process (GO:0072593), and nitric oxide biosynthetic process (GO:0006809); (3) blood circulation and blood pressure control: vasoconstriction (GO:0042310), negative regulation of blood vessel diameter (GO:0097756), regulation of blood pressure (GO:0008217), negative regulation of systemic arterial blood pressure (GO:0003085), and positive regulation of blood circulation (GO:1903524); (4) cellular metabolic process: cAMP-mediated signaling (GO:0019933), adenylate cyclase-modulating G protein-coupled receptor signaling pathway (GO:0007188), cholesterol metabolic process (GO:0008203), glucose transmembrane transport (GO:1904659), and fatty acid catabolic process (GO:0009062). These biological functions of potential targets of TwHF may represent implicated mechanisms in the treatment of DKD.

### 3.4. Pathway of Compound-DKD PPI Network

**Figure 5:**
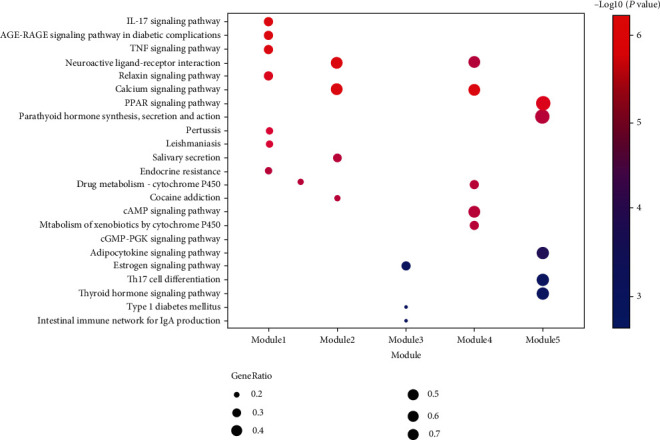
KEGG pathway analysis of 5 clusters. The color of nodes was described in a gradient from blue to red according to the descending order of the *P* value. GeneRatio equaled to gene count enriched in pathway/total gene count enriched in the cluster.

**Figure 6:**
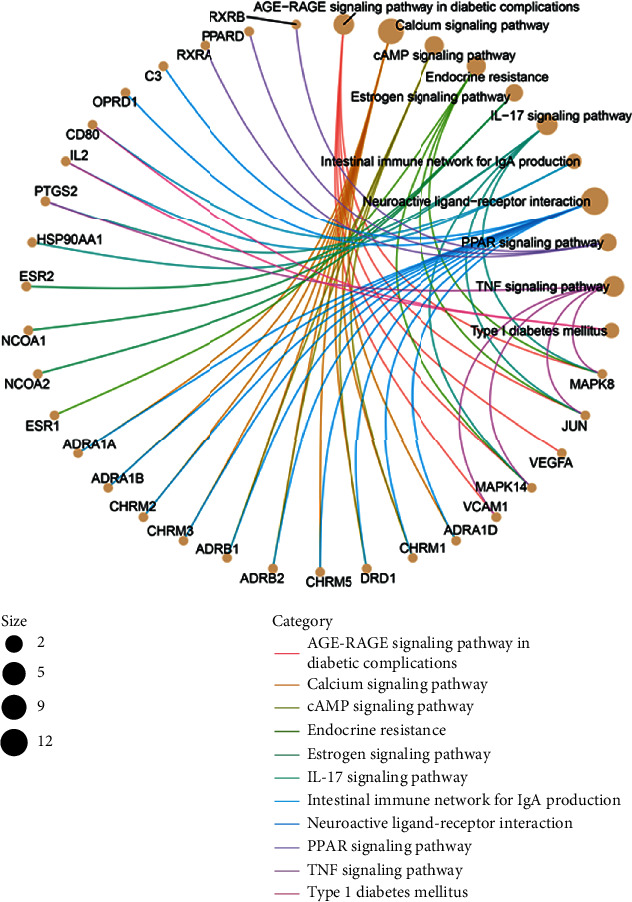
The potential key signaling pathways and involved genes of TwHF for treating DKD.

To further reveal the potential mechanism of the TwHF on DKD, KEGG pathway enrichment analysis was conducted on the genes in the abovementioned five clusters. As shown in Table S4 and Figure 5, there are some KEGG pathways in relation to DKD were enriched to five clusters. Cluster 1 mainly enriched in AGE-RAGE signaling pathway, IL-17 signaling pathway, TNF signaling pathway, and endocrine resistance. Cluster 2 gets neuroactive ligand-receptor interaction and calcium signaling pathway. Cluster 3 includes estrogen signaling pathway and type 1 diabetes mellitus. Cluster 4 gets calcium signaling pathway. Cluster 5 contains PPAR signaling pathway. Key pathways related to DKD and involved genes were described in Figure 6.

The generation and accumulation of AGEs in various tissues are highly facilitated under diabetic conditions due to persistent hyperglycemia and increased insulin resistance. The kidney acts as a crucial target for AGE-mediated impairment as well as a contributor to increasing AGE that results from a decrease in renal function, by the clearance of AGE [41]. Interaction of AGEs with the receptor for AGEs (RAGE) provokes deleterious intracellular signaling cascades, activating oxidative stress, inflammatory, and fibrotic response in diabetic kidney tissues, ultimately leading to renal dysfunction [42]. Increase in AGE-RAGE interaction under diabetic conditions activates renal VEGFA expression among various in vitro and in vivo experimental models and further aggravates the release of inflammatory factors [31]. Treatment with RAGE-aptamer to block the binding of AGEs to RAGE resulted in decreased ROS production, albuminuria, and extracellular matrix accumulation, accompanying with increased NADPH oxidase activity in kidney tissues from diabetic rats [43]. Consistently, RAGE knockout in streptozotocin-induced diabetic mice showed significantly remission in mesangial expansion or glomerular basement membrane thickening, while mice administered with AGE-albumin developed NF-*κ*B activation, upregulated collagen IV, and glomerulosclerosis in kidney tissues [44]. In addition, studies suggest that AGE-RAGE axis stimulates renal inflammation through NF-*κ*B signaling pathway and the release of central proinflammatory cytokine TNF-*α*, and the interaction of AGE-RAGE and TNF signaling pathway could induce the excessive generation of superoxide in diabetes [45].

Chronic inflammation has a pivotal role in the development and progression of DKD. Tumor necrosis factor (TNF) functions as a critical cytokine in regulating a wide range of intracellular signal pathways including inflammation and immunity, which has been actively implicated in the renal pathophysiology and activated mainly by NF-*κ*B signaling pathway and MAPK cascade [46]. There is an increasing evidence that TNF-*α* generated from renal cells and infiltrating macrophages has shown the ability to modulate several mechanisms involved in the DKD development and progression, including promoting permeability of the glomerular basement membrane and glomerular vasoconstriction, decreasing intraglomerular blood flow [47]. Recent findings from clinical specimens with DKD demonstrated that TNF gene expression of renal tubule was associated with increased fibrosis and reduced GFR [48], and there is a close correlation between a decline in renal function and circulating TNF-*α* and its receptor (TNFR) [49]. Of note, it has been reported that multiglycoside of TwHF could inhibit the activation of p38 MAPK and NF-*κ*B signaling and suppress overexpression of proinflammatory cytokines (TNF-*α*, IL-1*β*, and TGF-*β*1) in kidney, which may contribute to the improvement in glomerulosclerosis within diabetic rats [13]. Another study revealed that the intervention of triptolide, a major active component of TwHF, alleviated DKD through maintaining Th1/Th2 cell balance accompanied by downregulated IFN-*γ* and TNF-*α* and upregulated IL-4 and IL-10 [50]. Furthermore, Ma et al. confirmed that IL-17 deficient mice developed a reduction in albuminuria and an improvement in glomerular damage and fibrosis under diabetic setting, implying the contributing role of IL-17 signaling in the pathogenesis of DKD [51]. The inhibitory effect of TwHF and its extracts on IL-17 has been identified in autoimmune diseases including psoriasis, ankylosing spondylitis, and rheumatoid arthritis [52–54], but not in DKD yet.

Emerging evidence suggests that calcium signaling pathway appears to be vital in the development of DKD. It has shown that the function of native kidney cells could be controlled by intracellular Ca2+ signaling [55]. High glucose-induced Ca^2+^ alternation in mesangial cells enhanced the deposition of extracellular matrix proteins and mesangial expansion, thereby leading to glomerular injury [56], and the albumin endocytosis in proximal tubular epithelial cells was Ca^2+^-dependent [56]. As for podocyte, studies have revealed that calcium influx in response to angiotensin II, ROS, and other factors under diabetic conditions resulted in podocyte hypertrophy and foot process effacement, which may contribute to albuminuria and progression to DKD [57]. Additionally, increased intracellular Ca2+ levels promoted LKB1-AMPK/PPAR*α* pathway in high glucose-treated glomerular endothelial cells and podocytes, thus attenuating oxidative stress and apoptosis [58].

Based on the degree value of each gene in compound target-DKD target network, VEGFA was considered as the core gene of TwHF against DKD. The most potent angiogenic factor, VEGFA, is a major contributor to the development of new vessels and is also identified as a proinflammatory cytokine [59]. Data obtained from BTBR ob/ob mice, streptozotocin-induced diabetic mice, and patients with DKD has demonstrated that VEGFA was upregulated under diabetic environment [36, 60, 61], and increased VEGFA was closely related to a variety of pathological abnormalities in DKD such as glomerular hypertrophy, foot process effacement, fibrosis, and albuminuria [31], implying the crucial role of VEGFA in the pathogenesis of DKD. There is a significant relationship between VEGFA and kidney inflammation in the settings of diabetes. Elevated serum VEGFA levels in DKD patients was associated with enhanced expression of inflammatory biomarkers [61], while pharmacologic inhibition of VEGFA attenuated kidney pathological damage and albuminuria through suppressing endothelial activation and reducing glomerular macrophage infiltration and TNF-*α* expression [36]. Lavoz et al. found that blockage of VEGFR2, the receptor of VEGFA, has shown beneficial effects in treating DKD by downregulating the expression of proinflammatory mediators such as MCP-1, IL-6, and IL-17a, thus improving tubulointerstitial inflammation and ultimately reversing diabetic kidney impairment [60]. Otherwise, VEGFA expression in adipose tissue has been reported to be correlated with the development of obesity and insulin resistance [59]. Therefore, the key mechanisms of TwHF against DKD might be to rugulate VEGFA overexpression and thereby suppress a cascade of proinflammatory factors and intracellular signaling, resulting in the remission of kidney damage under diabetic conditions. Studies have revealed that triptolide, a diterpenoid triepoxide from TwHF, might exert antiangiogenic effect on rheumatoid arthritis via inhibiting VEGF, TNF-*α*, and IL-17 [54], and further experiment needs to be conducted to investigate the specific pharmacological effect of TwHF on DKD.

DKD poses a major public-health burden globally attributed to the dramatically increasing prevalence of diabetes. Despite great efforts in looking for pharmacological strategies to control the disease based on the one-target one-drug paradigm, the incidence of ESRD continues to rise; thus, multiple component-multiple target pattern receives a large amount of attention recently. Tripterygium has been widely used among Chinese practitioners for thousands of years in treating DKD and exhibits remarkable effects in reducing albuminuria excretion. Clinical trials have confirmed that TwHF and its extracts are effective in treating DKD, including decreasing proteinuria and decrease in eGFR [62]. At the same time, our research indicated that TwHF might modulate lipid homeostasis and insulin resistance under diabetic environment, which provided a novel explanation for elucidating the potential mechanisms of TwHF on DKD, but our hypotheses need further experimental validation. Notably, adverse events related to TwHF treatment including liver toxicity, infertility, and hematopoietic disorders have been reported in recent years [63] and received increasingly attention. Diterpenoids, alkaloids, and triterpenoids are the major toxic components of Tripterygium [64]. We analyzed top 20 compounds of TwHF against DKD according to the Comparative Toxicogenomics Database and found that there are 6 diterpenoids, 1 triterpenoid, and 0 alkaloids. However, the high-potential components kaempferol, beta-sitosterol, and stigmasterol in the treatment of DKD belong to flavonoids and phytosterols, respectively. Therefore, our study helps to identify the active components of TwHF against DKD as well as may be regarded as a good beginning point for designing new drugs with fewer side effects.

## 4. Conclusion

Our research is the first report to explain the active ingredients and mechanisms of TwHF against DKD using network pharmacology. A total of 51 active ingredients in TwHF were screened and hit by 88 potential targets related to DKD, and kaempferol and VEGFA were represented as the uppermost active ingredient and core gene of TwHF in treating DKD, respectively. The mechanisms of TwHF against DKD were associated with 5 functional clusters, and the key mechanisms of TwHF against DKD might be involved in the reduction of renal inflammation by downregulating VEGFA. Although the present study provided a methodological exploration for identifying potential active compound and pharmacological mechanism of TwHF on DKD, in vivo and in vitro experimental validation is required in the next step to support our findings.

## Figures and Tables

**Table 1:** Basic information of active components in TwHF.

Mol ID	Compound	MW	OB (%)	DL	Targets
MOL000422	Kaempferol	286.25	41.88	0.24	46
MOL000358	Beta-sitosterol	414.79	36.91	0.75	39
MOL000449	Stigmasterol	412.77	43.83	0.76	38
MOL003231	Triptoditerpenic acid B	328.49	40.02	0.36	33
MOL003196	Tryptophenolide	312.44	48.5	0.44	29
MOL003229	Triptinin B	314.46	34.73	0.32	29
MOL003184	81827-74-9	342.47	45.42	0.53	27
MOL003280	Triptonolide	326.42	49.51	0.49	27
MOL000296	Hederagenin	414.79	36.91	0.75	26
MOL003217	Isoxanthohumol	354.43	56.81	0.39	26
MOL005828	Nobiletin flavones	402.43	61.67	0.52	25
MOL003248	Triptonoterpene	300.48	48.57	0.28	24
MOL003185	(1R,4aR,10aS)-5-hydroxy-1-(hydroxymethyl)-7-isopropyl-8-methoxy-1,4a-dimethyl-4,9,10,10a-tetrahydro-3H-phenanthren-2-one	346.51	48.84	0.38	23
MOL003199	5,8-Dihydroxy-7-(4-hydroxy-5-methyl-coumarin-3)-coumarin	352.31	61.85	0.54	22
MOL003283	(2R,3R,4S)-4-(4-hydroxy-3-methoxy-phenyl)-7-methoxy-2,3-dimethylol-tetralin-6-ol	360.44	66.51	0.39	22

**Table 2:** Cluster of compound-DKD PPI network.

Cluster	Score	Nodes	Edges	Genes
1	11.29	15	79	AR, MAPK8, JUN, VEGFA, NR3C1, ESR1, MAPK14, NOS2, HSP90AA1, PGR, HMOX1, CD86, PTGS2, VCAM1, and CXCR4
2	6	13	36	SLC6A4, MAOA, MAOB, OPRD1, ADRA1D, CYP3A4, CHRM3, SLC6A2, CHRM2, ADRA1B, SLC6A3, ADRA1A, and C3
3	4.2	11	21	AHR, SELE, PLAU, NCOA2, PPARG, NCOA1, MMP1, IL2, CD80, ESR2, and KDR
4	3.56	10	16	ADH1C, GSTP1, CHRM1, DRD1, ACHE, CHRM5, PON1, ADRB2, ADRB1, and ADH1B
5	3.33	4	5	BCL2, RXRA, PPARD, and RXRB

## Data Availability

The data used to support the findings of this study are available from the corresponding author upon request.

## Supplementary Materials

Supplementary materials Table S1: Compound database of Tripterygium. Table S2: the GO and KEGG analyses on Triptoditerpenic acid B-DKD cotargets. Table S3: compound-DKD cotargets. Table S4: the GO enrichment analysis based on compound-DKD PPI network. Table S5: the KEGG pathway analysis based on compound-DKD PPI network. Table S6: the GO and KEGG analyses on Triptoditerpenic acid B and DKD cotargets. Table S7: the GO and KEGG analyses based on compound-DKD PPI network using WebGestalt. Figure S1: matching of target genes between DKD and TwHF.
