# Supplementary material for: A Network Pharmacology-Based Strategy for Unveiling the Mechanisms of Tripterygium Wilfordii Hook F against Diabetic Kidney Disease
Source: J Diabetes Res. 2020 Nov 20;2020:2421631. doi: 10.1155/2020/2421631 (PMC7695487; doi:10.1155/2020/2421631)
Supplement: Supplementary materials — Table S1: Compound database of Tripterygium. Table S2: the GO and KEGG analyses on Triptoditerpenic acid B-DKD cotargets. Table S3: compound-DKD cotargets. Table S4: the GO enrichment analysis based on compound-DKD PPI network. Table S5: the KEGG pathway analysis based on compound-DKD PPI network. Table S6: the GO and KEGG analyses on Triptoditerpenic acid B and DKD cotargets. Table S7: the GO and KEGG analyses based on compound-DKD PPI network using WebGestalt. Figure S1: matching of target genes between DKD and TwHF. [file 2421631.f1.docx]

Supplementary data

# A Network Pharmacology-Based Strategy for Unveiling the Mechanisms of Tripterygium wilfordii Hook F against Diabetic Kidney Disease

**Table S1: Compounds Database of Tripterygium**

| Mol ID | Compound | MW | OB(%) | DL | Targets |
| --- | --- | --- | --- | --- | --- |
| MOL000422 | kaempferol | 286.25 | 41.88 | 0.24 | 46 |
| MOL000358 | beta-sitosterol | 414.79 | 36.91 | 0.75 | 39 |
| MOL000449 | Stigmasterol | 412.77 | 43.83 | 0.76 | 38 |
| MOL003231 | Triptoditerpenic acid B | 328.49 | 40.02 | 0.36 | 33 |
| MOL003196 | Tryptophenolide | 312.44 | 48.5 | 0.44 | 29 |
| MOL003229 | Triptinin B | 314.46 | 34.73 | 0.32 | 29 |
| MOL003184 | 81827-74-9 | 342.47 | 45.42 | 0.53 | 27 |
| MOL003280 | TRIPTONOLIDE | 326.42 | 49.51 | 0.49 | 27 |
| MOL000296 | hederagenin | 414.79 | 36.91 | 0.75 | 26 |
| MOL003217 | Isoxanthohumol | 354.43 | 56.81 | 0.39 | 26 |
| MOL005828 | nobiletin | 402.43 | 61.67 | 0.52 | 25 |
| MOL003248 | Triptonoterpene | 300.48 | 48.57 | 0.28 | 24 |
| MOL003185 | (1R,4aR,10aS)-5-hydroxy-1-(hydroxymethyl)-7-isopropyl-8-methoxy-1,4a-dimethyl-4,9,10,10a-tetrahydro-3H-phenanthren-2-one | 346.51 | 48.84 | 0.38 | 23 |
| MOL003199 | 5,8-Dihydroxy-7-(4-hydroxy-5-methyl-coumarin-3)-coumarin | 352.31 | 61.85 | 0.54 | 22 |
| MOL003283 | (2R,3R,4S)-4-(4-hydroxy-3-methoxy-phenyl)-7-methoxy-2,3-dimethylol-tetralin-6-ol | 360.44 | 66.51 | 0.39 | 22 |
| MOL002058 | 40957-99-1 | 388.45 | 57.2 | 0.62 | 20 |
| MOL003245 | Triptonoditerpenic acid | 344.49 | 42.56 | 0.39 | 19 |
| MOL009386 | 3,3'-bis-(3,4-dihydro-4-hydroxy-6-methoxy)-2H-1-benzopyran | 358.42 | 52.11 | 0.54 | 19 |
| MOL003182 | (+)-Medioresinol di-O-beta-D-glucopyranoside_qt | 388.45 | 60.69 | 0.62 | 16 |
| MOL003187 | triptolide | 360.44 | 51.29 | 0.68 | 15 |
| MOL004443 | Zhebeiresinol | 280.3 | 58.72 | 0.19 | 14 |
| MOL007415 | [(2S)-2-[[(2S)-2-(benzoylamino)-3-phenylpropanoyl]amino]-3-phenylpropyl] acetate | 444.57 | 58.02 | 0.52 | 7 |
| MOL003209 | Celallocinnine | 405.59 | 83.47 | 0.59 | 6 |
| MOL003225 | Hypodiolide A | 318.5 | 76.13 | 0.49 | 4 |
| MOL003266 | 21-Hydroxy-30-norhopan-22-one | 428.77 | 34.11 | 0.77 | 4 |
| MOL000211 | Mairin | 456.78 | 55.38 | 0.78 | 4 |
| MOL003208 | Celafurine | 369.51 | 72.94 | 0.44 | 3 |
| MOL003224 | Tripdiotolnide | 360.44 | 56.4 | 0.67 | 3 |
| MOL011169 | Peroxyergosterol | 428.72 | 44.39 | 0.82 | 3 |
| MOL003189 | WILFORLIDE A | 486.81 | 35.66 | 0.72 | 2 |
| MOL007535 | (5S,8S,9S,10R,13R,14S,17R)-17-[(1R,4R)-4-ethyl-1,5-dimethylhexyl]-10,13-dimethyl-2,4,5,7,8,9,11,12,14,15,16,17-dodecahydro-1H-cyclopenta[a]phenanthrene-3,6-dione | 428.77 | 33.12 | 0.79 | 2 |
| MOL003232 | Triptofordin B1 | 478.63 | 39.55 | 0.84 | 1 |
| MOL003278 | salaspermic acid | 472.78 | 32.19 | 0.63 | 1 |
| MOL003188 | Tripchlorolide | 396.9 | 78.72 | 0.72 | 0 |
| MOL003192 | Triptonide | 344.39 | 67.66 | 0.7 | 0 |
| MOL003198 | 5 alpha-Benzoyl-4 alpha-hydroxy-1 beta,8 alpha-dinicotinoyl-dihydro-agarofuran | 600.72 | 35.26 | 0.72 | 0 |
| MOL003206 | Canin | 278.33 | 77.41 | 0.33 | 0 |
| MOL003210 | Celapanine | 569.66 | 30.18 | 0.82 | 0 |
| MOL003211 | Celaxanthin | 550.94 | 47.37 | 0.58 | 0 |
| MOL003222 | Salazinic acid | 402.33 | 36.34 | 0.76 | 0 |
| MOL003233 | Triptofordin B2 | 608.69 | 107.71 | 0.76 | 0 |
| MOL003234 | Triptofordin C2 | 610.71 | 30.16 | 0.76 | 0 |
| MOL003235 | Triptofordin D1 | 606.72 | 32 | 0.75 | 0 |
| MOL003236 | Triptofordin D2 | 650.78 | 30.38 | 0.69 | 0 |
| MOL003238 | Triptofordin F1 | 694.79 | 33.91 | 0.6 | 0 |
| MOL003239 | Triptofordin F2 | 668.75 | 33.62 | 0.67 | 0 |
| MOL003241 | Triptofordin F4 | 652.75 | 31.37 | 0.67 | 0 |
| MOL003242 | Triptofordinine A2 | 741.85 | 30.78 | 0.47 | 0 |
| MOL003244 | Triptonide | 358.42 | 68.45 | 0.68 | 0 |
| MOL003267 | Wilformine | 805.86 | 46.32 | 0.2 | 0 |
| MOL003279 | 99694-86-7 | 376.44 | 75.23 | 0.66 | 0 |

**Table S2: The GO and KEGG analysis on Triptoditerpenic acid B-DKD co targets**

|  | ID | Description | Count | *P* value | Gene |
| --- | --- | --- | --- | --- | --- |
| **GO** | GO:0009725 | response to hormone | 18 | 2.78E-11 | NOS2;CHRM3;CHRM1;ESR1;AR;CHRM5;PTGS2;CA2;RXRA;ADRA1A;PGR;CHRM2;ESR2;NR3C1;RXRB;CCNA2;NCOA2;NCOA1 |
|  | GO:0009719 | response to endogenous stimulus | 21 | 2.78E-11 | NOS2;CHRM3;CHRM1;ESR1;AR;CHRM5;PTGS2;CA2;RXRA;ADRA1A;PGR;CHRM2;ADRB2;OPRM1;ESR2;NR3C1;GSK3B;RXRB;CCNA2;NCOA2;NCOA1 |
|  | GO:0071495 | cellular response to endogenous stimulus | 19 | 2.00E-10 | CHRM3;CHRM1;ESR1;AR;CHRM5;PTGS2;CA2;RXRA;PGR;CHRM2;ADRB2;OPRM1;ESR2;NR3C1;GSK3B;RXRB;CCNA2;NCOA2;NCOA1 |
|  | GO:0007267 | cell-cell signaling | 20 | 2.00E-10 | NOS2;CHRM3;CHRM1;ESR1;AR;SCN5A;CHRM5;PTGS2;CA2;ACHE;ADRA1A;PGR;CHRM2;ADRA1B;ADRB2;ADRA1D;OPRM1;ESR2;DPP4;GSK3B |
|  | GO:0032870 | cellular response to hormone stimulus | 15 | 4.48E-10 | CHRM3;CHRM1;ESR1;AR;CHRM5;CA2;RXRA;PGR;CHRM2;ESR2;NR3C1;RXRB;CCNA2;NCOA2;NCOA1 |
|  | GO:0007188 | adenylate cyclase-modulating G protein-coupled receptor signaling pathway | 10 | 5.76E-09 | CHRM3;CHRM1;CHRM5;OPRD1;ADRA1A;CHRM2;ADRA1B;ADRB2;ADRA1D;OPRM1 |
|  | GO:0007187 | G protein-coupled receptor signaling pathway, coupled to cyclic nucleotide second messenger | 10 | 1.92E-08 | CHRM3;CHRM1;CHRM5;OPRD1;ADRA1A;CHRM2;ADRA1B;ADRB2;ADRA1D;OPRM1 |
|  | GO:0035690 | cellular response to drug | 11 | 1.92E-08 | NOS2;CHRM3;KCNH2;CHRM1;CHRM5;PTGS2;CHRM2;OPRM1;NR3C1;CCNA2;NCOA1 |
|  | GO:0048545 | response to steroid hormone | 11 | 5.33E-08 | ESR1;AR;PTGS2;CA2;RXRA;PGR;ESR2;NR3C1;RXRB;NCOA2;NCOA1 |
|  | GO:0033993 | response to lipid | 14 | 1.61E-07 | NOS2;ESR1;AR;PTGS2;CA2;RXRA;PGR;OPRM1;ESR2;NR3C1;RXRB;CCNA2;NCOA2;NCOA1 |
| **KEGG** | hsa04080 | Neuroactive ligand-receptor interaction | 12 | 1.13E-07 | CHRM3;CHRM1;CHRM5;OPRD1;ADRA1A;CHRM2;ADRA1B;ADRB2;ADRA1D;OPRM1;NR3C1;PRSS1 |
|  | hsa04020 | Calcium signaling pathway | 9 | 4.92E-06 | NOS2;CHRM3;CHRM1;CHRM5;ADRA1A;CHRM2;ADRA1B;ADRB2;ADRA1D |
|  | hsa04915 | Estrogen signaling pathway | 7 | 1.11E-04 | ESR1;PGR;OPRM1;ESR2;HSP90AA1;NCOA2;NCOA1 |
|  | hsa04919 | Thyroid hormone signaling pathway | 6 | 5.13E-04 | ESR1;RXRA;GSK3B;RXRB;NCOA2;NCOA1 |
|  | hsa05200 | Pathways in cancer | 10 | 0.001576 | NOS2;ESR1;AR;PTGS2;RXRA;ESR2;GSK3B;HSP90AA1;RXRB;NCOA1 |
|  | hsa04970 | Salivary secretion | 5 | 0.001576 | CHRM3;ADRA1A;ADRA1B;ADRB2;ADRA1D |
|  | hsa04725 | Cholinergic synapse | 5 | 0.00387 | CHRM3;CHRM1;CHRM5;ACHE;CHRM2 |
|  | hsa04261 | Adrenergic signaling in cardiomyocytes | 5 | 0.010832 | SCN5A;ADRA1A;ADRA1B;ADRB2;ADRA1D |
|  | hsa05224 | Breast cancer | 5 | 0.010832 | ESR1;PGR;ESR2;GSK3B;NCOA1 |
|  | hsa04022 | cGMP-PKG signaling pathway | 5 | 0.015156 | OPRD1;ADRA1A;ADRA1B;ADRB2;ADRA1D |

**Table S3: Compound-DKD Co-targets**

| No. | Symbol name | Gene name | No. | Symbol name | Gene name |
| --- | --- | --- | --- | --- | --- |
| 1 | PTGS1 | Prostaglandin G/H synthase 1 | 45 | IFNGR1 | Interferon gamma |
| 2 | CHRM3 | Muscarinic acetylcholine receptor M3 | 46 | JUN | Transcription factor AP-1 |
| 3 | CHRM1 | Muscarinic acetylcholine receptor M1 | 47 | TP63 | Cellular tumor antigen p53 |
| 4 | ESR1 | Estrogen receptor1 | 48 | C3 | Complement C3 |
| 5 | AR | Androgen receptor | 49 | MAPK8 | Mitogen-activated protein kinase 8 |
| 6 | SCN5A | Sodium channel protein type 5 subunit alpha | 50 | VEGFA | Vascular endothelial growth factor A |
| 7 | PPARG | Peroxisome proliferator activated receptor gamma | 51 | IL2 | Interleukin-2 |
| 8 | PTGS2 | Prostaglandin G/H synthase 2 | 52 | DRD1 | Dopamine D1 receptor |
| 9 | CA2 | Carbonic anhydrase II | 53 | CDK2 | Cell division protein kinase 2 |
| 10 | RXRA | Retinoic acid receptor RXR-alpha | 54 | KDR | Vascular endothelial growth factor receptor 2 |
| 11 | ACHE | Acetylcholinesterase | 55 | PYGM | Glycogen phosphorylase, muscle form |
| 12 | SLC6A2 | Sodium-dependent noradrenaline transporter | 56 | MAPK14 | Mitogen-activated protein kinase 14 |
| 13 | PGR | Progesterone receptor | 57 | CCNA2 | Cyclin-A2 |
| 14 | CHRM2 | Muscarinic acetylcholine receptor M2 | 58 | PPARD | Peroxisome proliferator activated receptor delta |
| 15 | ADRA1B | Alpha-1B adrenergic receptor | 59 | CHEK1 | Serine/threonine-protein kinase Chk1 |
| 16 | NR3C1 | Glucocorticoid receptor | 60 | NR3C2 | Mineralocorticoid receptor |
| 17 | GABRA1 | Gamma-aminobutyric acid receptor subunit alpha-1 | 61 | RXRB | Retinoic acid receptor RXR-beta |
| 18 | DPP4 | Dipeptidyl peptidase IV | 62 | PON1 | Serum paraoxonase/arylesterase 1 |
| 19 | ADH1B | Alcohol dehydrogenase 1B | 63 | MAP2 | Microtubule-associated protein 2 |
| 20 | ADH1C | Alcohol dehydrogenase 1C | 64 | SLC6A4 | Sodium-dependent serotonin transporter |
| 21 | PRSS1 | Trypsin-1 | 65 | INSRR | Insulin receptor |
| 22 | GRIA2 | Glutamate receptor 2 | 66 | ALOX5 | Arachidonate 5-lipoxygenase |
| 23 | NCOA2 | Nuclear receptor coactivator 2 | 67 | AKR1C3 | Aldo-keto reductase family 1 member C3 |
| 24 | NOS2 | Nitric oxide synthase, inducible | 68 | MMP1 | Interstitial collagenase |
| 25 | KCNH2 | Potassium voltage-gated channel subfamily H member 2 | 69 | SELE | E-selectin |
| 26 | F7 | Coagulation factor VII | 70 | CDK1 | Cell division control protein 2 homolog |
| 27 | ESR2 | Estrogen receptor2 | 71 | VCAM1 | Vascular cell adhesion protein 1 |
| 28 | GSK3B | Glycogen synthase kinase-3 beta | 72 | CYP3A4 | Cytochrome P450 3A4 |
| 29 | HSP90AA1 | Heat shock protein HSP 90 | 73 | CYP1A2 | Cytochrome P450 1A2 |
| 30 | CALM1 | Calmodulin | 74 | GSTP1 | Glutathione S-transferase P |
| 31 | CHRM5 | Muscarinic acetylcholine receptor M5 | 75 | HMOX1 | Heme oxygenase 1 |
| 32 | OPRD1 | Delta-type opioid receptor | 76 | GSTM1 | Glutathione S-transferase Mu 1 |
| 33 | ADRB2 | Beta-2 adrenergic receptor | 77 | AHR | Aryl hydrocarbon receptor |
| 34 | OPRM1 | Mu-type opioid receptor | 78 | GSTM2 | Glutathione S-transferase Mu 2 |
| 35 | NCOA1 | Nuclear receptor coactivator 1 | 79 | PPP3CA | Serine/threonine-protein phosphatase 2B catalytic subunit alpha isoform |
| 36 | ADRA1A | Alpha-1A adrenergic receptor | 80 | ADRB1 | Beta-1 adrenergic receptor |
| 37 | ADRA1D | Alpha-1D adrenergic receptor | 81 | ADRA2A | Alpha-2A adrenergic receptor |
| 38 | BCL2 | Apoptosis regulator Bcl-2 | 82 | SLC6A3 | Sodium-dependent dopamine transporter |
| 39 | CD1A | T-cell surface glycoprotein CD1a | 83 | AKR1B1 | Aldose reductase |
| 40 | TNFSF15 | Tumor necrosis factor | 84 | LTA4H | Leukotriene A-4 hydrolase |
| 41 | CD86 | T-lymphocyte activation antigen CD86 | 85 | MAOB | Amine oxidase [flavin-containing] B |
| 42 | CD80 | T-lymphocyte activation antigen CD80 | 86 | MAOA | Amine oxidase [flavin-containing] A |
| 43 | PLAU | Urokinase-type plasminogen activator | 87 | CTRB1 | Chymotrypsinogen B |
| 44 | CXCR4 | C-X-C chemokine receptor type 4 | 88 | PLA2G4A | Cytosolic phospholipase A2 |

**Table S4: The GO enrichment analysis based on compound-DKD PPI network**

| Module | ID | Description | Count | *P* value | Gene |
| --- | --- | --- | --- | --- | --- |
| Cluster1 | GO:0032496 | response to lipopolysaccharide | 7 | 2.88E-09 | MAPK8/JUN/MAPK14/NOS2/CD86/PTGS2/VCAM1 |
|  | GO:0002237 | response to molecule of bacterial origin | 7 | 3.77E-09 | MAPK8/JUN/MAPK14/NOS2/CD86/PTGS2/VCAM1 |
|  | GO:0051090 | regulation of DNA-binding transcription factor activity | 7 | 1.85E-08 | AR/MAPK8/JUN/VEGFA/ESR1/MAPK14/HMOX1 |
|  | GO:0060135 | maternal process involved in female pregnancy | 4 | 1.67E-07 | AR/ESR1/PGR/PTGS2 |
|  | GO:0001666 | response to hypoxia | 6 | 2.10E-07 | VEGFA/NOS2/HMOX1/PTGS2/VCAM1/CXCR4 |
|  | GO:0036293 | response to decreased oxygen levels | 6 | 2.50E-07 | VEGFA/NOS2/HMOX1/PTGS2/VCAM1/CXCR4 |
|  | GO:0060749 | mammary gland alveolus development | 3 | 3.40E-07 | AR/VEGFA/ESR1 |
|  | GO:0061377 | mammary gland lobule development | 3 | 3.40E-07 | AR/VEGFA/ESR1 |
|  | GO:0070482 | response to oxygen levels | 6 | 3.62E-07 | VEGFA/NOS2/HMOX1/PTGS2/VCAM1/CXCR4 |
|  | GO:0002064 | epithelial cell development | 5 | 4.38E-07 | AR/VEGFA/ESR1/PGR/CXCR4 |
|  | GO:0009612 | response to mechanical stimulus | 5 | 4.70E-07 | MAPK8/JUN/MAPK14/PTGS2/CXCR4 |
|  | GO:0048608 | reproductive structure development | 6 | 6.13E-07 | AR/VEGFA/ESR1/MAPK14/PGR/PTGS2 |
|  | GO:0048732 | gland development | 6 | 6.39E-07 | AR/JUN/VEGFA/ESR1/PGR/HMOX1 |
|  | GO:0050673 | epithelial cell proliferation | 6 | 6.39E-07 | AR/JUN/VEGFA/ESR1/PGR/HMOX1 |
|  | GO:0061458 | reproductive system development | 6 | 6.39E-07 | AR/VEGFA/ESR1/MAPK14/PGR/PTGS2 |
|  | GO:0031281 | positive regulation of cyclase activity | 3 | 6.40E-07 | MAPK8/MAPK14/NOS2 |
|  | GO:0060444 | branching involved in mammary gland duct morphogenesis | 3 | 9.55E-07 | AR/ESR1/PGR |
|  | GO:0006352 | DNA-templated transcription, initiation | 5 | 1.09E-06 | AR/JUN/NR3C1/ESR1/PGR |
|  | GO:0031668 | cellular response to extracellular stimulus | 5 | 1.57E-06 | MAPK8/JUN/HMOX1/PTGS2/VCAM1 |
|  | GO:0060603 | mammary gland duct morphogenesis | 3 | 2.26E-06 | AR/ESR1/PGR |
| Cluster2 | GO:0001505 | regulation of neurotransmitter levels | 7 | 1.29E-09 | SLC6A4/MAOA/MAOB/SLC6A2/CHRM2/SLC6A3/ADRA1A |
|  | GO:0007188 | adenylate cyclase-modulating G protein-coupled receptor signaling pathway | 6 | 4.12E-09 | OPRD1/ADRA1D/CHRM3/CHRM2/ADRA1B/ADRA1A |
|  | GO:0007187 | G protein-coupled receptor signaling pathway, coupled to cyclic nucleotide second messenger | 6 | 9.92E-09 | OPRD1/ADRA1D/CHRM3/CHRM2/ADRA1B/ADRA1A |
|  | GO:0006836 | neurotransmitter transport | 6 | 1.33E-08 | SLC6A4/MAOB/SLC6A2/CHRM2/SLC6A3/ADRA1A |
|  | GO:0019336 | phenol-containing compound catabolic process | 3 | 5.78E-08 | MAOA/MAOB/SLC6A3 |
|  | GO:0006940 | regulation of smooth muscle contraction | 4 | 9.34E-08 | CHRM3/CHRM2/ADRA1B/ADRA1A |
|  | GO:0042310 | vasoconstriction | 4 | 1.76E-07 | SLC6A4/CHRM3/ADRA1B/ADRA1A |
|  | GO:0097756 | negative regulation of blood vessel diameter | 4 | 2.64E-07 | SLC6A4/CHRM3/ADRA1B/ADRA1A |
|  | GO:0015844 | monoamine transport | 4 | 3.49E-07 | SLC6A4/MAOB/SLC6A2/SLC6A3 |
|  | GO:0007200 | phospholipase C-activating G protein-coupled receptor signaling pathway | 4 | 5.34E-07 | OPRD1/CHRM2/ADRA1B/ADRA1A |
|  | GO:0015696 | ammonium transport | 4 | 7.27E-07 | SLC6A4/MAOB/SLC6A2/SLC6A3 |
|  | GO:0006939 | smooth muscle contraction | 4 | 7.83E-07 | CHRM3/CHRM2/ADRA1B/ADRA1A |
|  | GO:0071880 | adenylate cyclase-activating adrenergic receptor signaling pathway | 3 | 1.17E-06 | ADRA1D/ADRA1B/ADRA1A |
|  | GO:0045907 | positive regulation of vasoconstriction | 3 | 1.29E-06 | CHRM3/ADRA1B/ADRA1A |
|  | GO:0045987 | positive regulation of smooth muscle contraction | 3 | 1.29E-06 | CHRM3/ADRA1B/ADRA1A |
|  | GO:0071875 | adrenergic receptor signaling pathway | 3 | 1.56E-06 | ADRA1D/ADRA1B/ADRA1A |
|  | GO:0051588 | regulation of neurotransmitter transport | 4 | 2.00E-06 | SLC6A4/MAOB/CHRM2/ADRA1A |
|  | GO:0042737 | drug catabolic process | 4 | 2.05E-06 | MAOA/MAOB/CYP3A4/SLC6A3 |
|  | GO:0035296 | regulation of tube diameter | 4 | 2.24E-06 | SLC6A4/CHRM3/ADRA1B/ADRA1A |
|  | GO:0050880 | regulation of blood vessel size | 4 | 2.24E-06 | SLC6A4/CHRM3/ADRA1B/ADRA1A |
| Cluster3 | GO:1901654 | response to ketone | 4 | 3.45E-06 | AHR/NCOA2/PPARG/NCOA1 |
|  | GO:0030522 | intracellular receptor signaling pathway | 4 | 1.50E-05 | AHR/PPARG/NCOA1/ESR2 |
|  | GO:0048511 | rhythmic process | 4 | 1.85E-05 | AHR/NCOA2/PPARG/NCOA1 |
|  | GO:0048545 | response to steroid hormone | 4 | 5.24E-05 | NCOA2/PPARG/NCOA1/ESR2 |
|  | GO:0015718 | monocarboxylic acid transport | 3 | 0.000101 | NCOA2/PPARG/NCOA1 |
|  | GO:0015721 | bile acid and bile salt transport | 2 | 0.00011 | NCOA2/NCOA1 |
|  | GO:0043401 | steroid hormone mediated signaling pathway | 3 | 0.000147 | PPARG/NCOA1/ESR2 |
|  | GO:0045622 | regulation of T-helper cell differentiation | 2 | 0.000186 | IL2/CD80 |
|  | GO:0007623 | circadian rhythm | 3 | 0.000211 | AHR/NCOA2/PPARG |
|  | GO:0050670 | regulation of lymphocyte proliferation | 3 | 0.000211 | AHR/IL2/CD80 |
|  | GO:0032944 | regulation of mononuclear cell proliferation | 3 | 0.000214 | AHR/IL2/CD80 |
|  | GO:0070663 | regulation of leukocyte proliferation | 3 | 0.000255 | AHR/IL2/CD80 |
|  | GO:0097305 | response to alcohol | 3 | 0.000294 | AHR/PPARG/IL2 |
|  | GO:0009755 | hormone-mediated signaling pathway | 3 | 0.000302 | PPARG/NCOA1/ESR2 |
|  | GO:0032570 | response to progesterone | 2 | 0.000308 | NCOA2/NCOA1 |
|  | GO:0043370 | regulation of CD4-positive, alpha-beta T cell differentiation | 2 | 0.000336 | IL2/CD80 |
|  | GO:0071383 | cellular response to steroid hormone stimulus | 3 | 0.000362 | PPARG/NCOA1/ESR2 |
|  | GO:0015850 | organic hydroxy compound transport | 3 | 0.000415 | NCOA2/PPARG/NCOA1 |
|  | GO:0046651 | lymphocyte proliferation | 3 | 0.000463 | AHR/IL2/CD80 |
|  | GO:0032943 | mononuclear cell proliferation | 3 | 0.000473 | AHR/IL2/CD80 |
| Cluster4 | GO:0007188 | adenylate cyclase-modulating G protein-coupled receptor signaling pathway | 5 | 5.33E-08 | CHRM1/DRD1/CHRM5/ADRB2/ADRB1 |
|  | GO:0007187 | G protein-coupled receptor signaling pathway, coupled to cyclic nucleotide second messenger | 5 | 1.11E-07 | CHRM1/DRD1/CHRM5/ADRB2/ADRB1 |
|  | GO:0042311 | vasodilation | 3 | 5.44E-07 | DRD1/ADRB2/ADRB1 |
|  | GO:0007190 | activation of adenylate cyclase activity | 3 | 9.24E-07 | DRD1/ADRB2/ADRB1 |
|  | GO:0001659 | temperature homeostasis | 4 | 1.43E-06 | DRD1/ACHE/ADRB2/ADRB1 |
|  | GO:0097755 | positive regulation of blood vessel diameter | 3 | 3.54E-06 | DRD1/ADRB2/ADRB1 |
|  | GO:0071242 | cellular response to ammonium ion | 3 | 5.45E-06 | CHRM1/DRD1/CHRM5 |
|  | GO:0040015 | negative regulation of multicellular organism growth | 2 | 1.42E-05 | ADRB2/ADRB1 |
|  | GO:0120162 | positive regulation of cold-induced thermogenesis | 3 | 1.59E-05 | ACHE/ADRB2/ADRB1 |
|  | GO:0002024 | diet induced thermogenesis | 2 | 1.70E-05 | ADRB2/ADRB1 |
|  | GO:0006069 | ethanol oxidation | 2 | 1.70E-05 | ADH1C/ADH1B |
|  | GO:0031649 | heat generation | 2 | 3.50E-05 | ADRB2/ADRB1 |
|  | GO:0060359 | response to ammonium ion | 3 | 4.28E-05 | CHRM1/DRD1/CHRM5 |
|  | GO:0003085 | negative regulation of systemic arterial blood pressure | 2 | 4.39E-05 | ADRB2/ADRB1 |
|  | GO:0007213 | G protein-coupled acetylcholine receptor signaling pathway | 2 | 4.39E-05 | CHRM1/CHRM5 |
|  | GO:0007189 | adenylate cyclase-activating G protein-coupled receptor signaling pathway | 3 | 4.66E-05 | DRD1/ADRB2/ADRB1 |
|  | GO:0060078 | regulation of postsynaptic membrane potential | 3 | 4.77E-05 | CHRM1/ADRB2/ADRB1 |
|  | GO:0035296 | regulation of tube diameter | 3 | 5.08E-05 | DRD1/ADRB2/ADRB1 |
|  | GO:0050880 | regulation of blood vessel size | 3 | 5.08E-05 | DRD1/ADRB2/ADRB1 |
|  | GO:0097746 | regulation of blood vessel diameter | 3 | 5.08E-05 | DRD1/ADRB2/ADRB1 |
| Cluster5 | GO:0048545 | response to steroid hormone | 4 | 1.78E-07 | BCL2/RXRA/PPARD/RXRB |
|  | GO:0043401 | steroid hormone mediated signaling pathway | 3 | 3.74E-06 | RXRA/PPARD/RXRB |
|  | GO:0006367 | transcription initiation from RNA polymerase II promoter | 3 | 3.99E-06 | RXRA/PPARD/RXRB |
|  | GO:0007565 | female pregnancy | 3 | 4.25E-06 | BCL2/RXRA/PPARD |
|  | GO:0044706 | multi-multicellular organism process | 3 | 6.58E-06 | BCL2/RXRA/PPARD |
|  | GO:0009755 | hormone-mediated signaling pathway | 3 | 7.80E-06 | RXRA/PPARD/RXRB |
|  | GO:0006352 | DNA-templated transcription, initiation | 3 | 9.28E-06 | RXRA/PPARD/RXRB |
|  | GO:0071383 | cellular response to steroid hormone stimulus | 3 | 9.40E-06 | RXRA/PPARD/RXRB |
|  | GO:0030522 | intracellular receptor signaling pathway | 3 | 1.32E-05 | RXRA/PPARD/RXRB |
|  | GO:0048384 | retinoic acid receptor signaling pathway | 2 | 1.49E-05 | RXRA/RXRB |
|  | GO:0001893 | maternal placenta development | 2 | 2.04E-05 | RXRA/PPARD |
|  | GO:0007566 | embryo implantation | 2 | 4.37E-05 | RXRA/PPARD |
|  | GO:0048608 | reproductive structure development | 3 | 4.80E-05 | BCL2/RXRA/PPARD |
|  | GO:0061458 | reproductive system development | 3 | 4.90E-05 | BCL2/RXRA/PPARD |
|  | GO:0060135 | maternal process involved in female pregnancy | 2 | 6.91E-05 | RXRA/PPARD |
|  | GO:0014855 | striated muscle cell proliferation | 2 | 0.000111 | RXRA/PPARD |
|  | GO:0014910 | regulation of smooth muscle cell migration | 2 | 0.000119 | BCL2/PPARD |
|  | GO:0014909 | smooth muscle cell migration | 2 | 0.00014 | BCL2/PPARD |
|  | GO:0014812 | muscle cell migration | 2 | 0.000183 | BCL2/PPARD |
|  | GO:0032526 | response to retinoic acid | 2 | 0.000197 | RXRA/RXRB |

Top 20 biological processes in each cluster.

**Table S5: The KEGG pathway analysis based on compound-DKD PPI network** *^a,b,c,d,e^*

1. The detailed information of Cluster 1

| Pathway ID | Pathway name | Count | *P* value | Gene |
| --- | --- | --- | --- | --- |
| hsa04657 | IL-17 signaling pathway | 5 | 8.33E-07 | MAPK8/JUN/MAPK14/HSP90AA1/PTGS2 |
| hsa04933 | AGE-RAGE signaling pathway in diabetic complications | 5 | 1.13E-06 | MAPK8/JUN/VEGFA/MAPK14/VCAM1 |
| hsa04668 | TNF signaling pathway | 5 | 1.99E-06 | MAPK8/JUN/MAPK14/PTGS2/VCAM1 |
| hsa04926 | Relaxin signaling pathway | 5 | 4.01E-06 | MAPK8/JUN/VEGFA/MAPK14/NOS2 |
| hsa05133 | Pertussis | 4 | 1.32E-05 | MAPK8/JUN/MAPK14/NOS2 |
| hsa05140 | Leishmaniasis | 4 | 1.39E-05 | JUN/MAPK14/NOS2/PTGS2 |
| hsa01522 | Endocrine resistance | 4 | 3.62E-05 | MAPK8/JUN/ESR1/MAPK14 |
| hsa04914 | Progesterone-mediated oocyte maturation | 4 | 3.77E-05 | MAPK8/MAPK14/HSP90AA1/PGR |
| hsa05142 | Chagas disease (American trypanosomiasis) | 4 | 4.24E-05 | MAPK8/JUN/MAPK14/NOS2 |
| hsa04620 | Toll-like receptor signaling pathway | 4 | 4.57E-05 | MAPK8/JUN/MAPK14/CD86 |
| hsa04625 | C-type lectin receptor signaling pathway | 4 | 4.57E-05 | MAPK8/JUN/MAPK14/PTGS2 |
| hsa04659 | Th17 cell differentiation | 4 | 5.11E-05 | MAPK8/JUN/MAPK14/HSP90AA1 |
| hsa04915 | Estrogen signaling pathway | 4 | 0.000138 | JUN/ESR1/HSP90AA1/PGR |
| hsa04370 | VEGF signaling pathway | 3 | 0.000209 | VEGFA/MAPK14/PTGS2 |
| hsa04917 | Prolactin signaling pathway | 3 | 0.000346 | MAPK8/ESR1/MAPK14 |
| hsa05120 | Epithelial cell signaling in Helicobacter pylori infection | 3 | 0.000346 | MAPK8/JUN/MAPK14 |
| hsa04621 | NOD-like receptor signaling pathway | 4 | 0.000392 | MAPK8/JUN/MAPK14/HSP90AA1 |
| hsa05132 | Salmonella infection | 4 | 0.00074 | MAPK8/JUN/MAPK14/HSP90AA1 |
| hsa04658 | Th1 and Th2 cell differentiation | 3 | 0.000773 | MAPK8/JUN/MAPK14 |
| hsa04912 | GnRH signaling pathway | 3 | 0.000798 | MAPK8/JUN/MAPK14 |
| hsa04660 | T cell receptor signaling pathway | 3 | 0.001105 | MAPK8/JUN/MAPK14 |
| hsa04066 | HIF-1 signaling pathway | 3 | 0.001266 | VEGFA/NOS2/HMOX1 |
| hsa05145 | Toxoplasmosis | 3 | 0.00137 | MAPK8/MAPK14/NOS2 |
| hsa04670 | Leukocyte transendothelial migration | 3 | 0.001405 | MAPK14/VCAM1/CXCR4 |
| hsa04722 | Neurotrophin signaling pathway | 3 | 0.001632 | MAPK8/JUN/MAPK14 |
| hsa05135 | Yersinia infection | 3 | 0.001671 | MAPK8/JUN/MAPK14 |
| hsa04114 | Oocyte meiosis | 3 | 0.002012 | AR/MAPK14/PGR |
| hsa04380 | Osteoclast differentiation | 3 | 0.002012 | MAPK8/JUN/MAPK14 |
| hsa04010 | MAPK signaling pathway | 4 | 0.002409 | MAPK8/JUN/VEGFA/MAPK14 |
| hsa04723 | Retrograde endocannabinoid signaling | 3 | 0.003044 | MAPK8/MAPK14/PTGS2 |
| hsa05161 | Hepatitis B | 3 | 0.003931 | MAPK8/JUN/MAPK14 |
| hsa04672 | Intestinal immune network for IgA production | 2 | 0.004322 | CD86/CXCR4 |
| hsa05152 | Tuberculosis | 3 | 0.005284 | MAPK8/MAPK14/NOS2 |
| hsa04510 | Focal adhesion | 3 | 0.006986 | MAPK8/JUN/VEGFA |
| hsa05130 | Pathogenic Escherichia coli infection | 3 | 0.007282 | MAPK8/JUN/MAPK14 |
| hsa04137 | Mitophagy - animal | 2 | 0.008187 | MAPK8/JUN |
| hsa04664 | Fc epsilon RI signaling pathway | 2 | 0.008187 | MAPK8/MAPK14 |
| hsa04622 | RIG-I-like receptor signaling pathway | 2 | 0.008659 | MAPK8/MAPK14 |
| hsa05131 | Shigellosis | 3 | 0.01155 | MAPK8/JUN/MAPK14 |
| hsa04012 | ErbB signaling pathway | 2 | 0.012577 | MAPK8/JUN |
| hsa04750 | Inflammatory mediator regulation of TRP channels | 2 | 0.017134 | MAPK8/MAPK14 |
| hsa04064 | NF-kappa B signaling pathway | 2 | 0.018453 | PTGS2/VCAM1 |

1. The detailed information of Cluster 2

| Pathway ID | Pathway name | Count | *P* value | Gene |
| --- | --- | --- | --- | --- |
| hsa04080 | Neuroactive ligand-receptor interaction | 7 | 5.74E-07 | OPRD1/ADRA1D/CHRM3/CHRM2/ADRA1B/ADRA1A/C3 |
| hsa04020 | Calcium signaling pathway | 5 | 1.28E-05 | ADRA1D/CHRM3/CHRM2/ADRA1B/ADRA1A |
| hsa04970 | Salivary secretion | 4 | 1.44E-05 | ADRA1D/CHRM3/ADRA1B/ADRA1A |
| hsa05030 | Cocaine addiction | 3 | 7.59E-05 | MAOA/MAOB/SLC6A3 |
| hsa04022 | cGMP-PKG signaling pathway | 4 | 0.000156 | OPRD1/ADRA1D/ADRA1B/ADRA1A |
| hsa05031 | Amphetamine addiction | 3 | 0.000211 | MAOA/MAOB/SLC6A3 |
| hsa00982 | Drug metabolism - cytochrome P450 | 3 | 0.00024 | MAOA/MAOB/CYP3A4 |
| hsa04721 | Synaptic vesicle cycle | 3 | 0.000304 | SLC6A4/SLC6A2/SLC6A3 |
| hsa00360 | Phenylalanine metabolism | 2 | 0.000387 | MAOA/MAOB |
| hsa00340 | Histidine metabolism | 2 | 0.000715 | MAOA/MAOB |
| hsa04726 | Serotonergic synapse | 3 | 0.000951 | SLC6A4/MAOA/MAOB |
| hsa04270 | Vascular smooth muscle contraction | 3 | 0.001418 | ADRA1D/ADRA1B/ADRA1A |
| hsa04728 | Dopaminergic synapse | 3 | 0.001418 | MAOA/MAOB/SLC6A3 |
| hsa00350 | Tyrosine metabolism | 2 | 0.001757 | MAOA/MAOB |
| hsa04261 | Adrenergic signaling in cardiomyocytes | 3 | 0.002009 | ADRA1D/ADRA1B/ADRA1A |
| hsa00260 | Glycine, serine and threonine metabolism | 2 | 0.002167 | MAOA/MAOB |
| hsa00380 | Tryptophan metabolism | 2 | 0.002387 | MAOA/MAOB |
| hsa00330 | Arginine and proline metabolism | 2 | 0.00337 | MAOA/MAOB |
| hsa05034 | Alcoholism | 3 | 0.003835 | MAOA/MAOB/SLC6A3 |
| hsa05012 | Parkinson disease | 3 | 0.008529 | MAOA/MAOB/SLC6A3 |
| hsa04080 | Neuroactive ligand-receptor interaction | 2 | 0.016348 | CHRM3/CHRM2 |

1. The detailed information of Cluster 3

| Pathway ID | Pathway name | Count | *P* value | Gene |
| --- | --- | --- | --- | --- |
| hsa04915 | Estrogen signaling pathway | 3 | 0.000956 | NCOA2/NCOA1/ESR2 |
| hsa04940 | Type I diabetes mellitus | 2 | 0.001777 | IL2/CD80 |
| hsa04672 | Intestinal immune network for IgA production | 2 | 0.002303 | IL2/CD80 |

1. The detailed information of Cluster 4

| Pathway ID | Pathway name | Count | *P* value | Gene |
| --- | --- | --- | --- | --- |
| hsa04020 | Calcium signaling pathway | 5 | 1.37E-06 | CHRM1/DRD1/CHRM5/ADRB2/ADRB1 |
| hsa04080 | Neuroactive ligand-receptor interaction | 5 | 2.22E-05 | CHRM1/DRD1/CHRM5/ADRB2/ADRB1 |
| hsa00982 | Drug metabolism - cytochrome P450 | 3 | 7.25E-05 | ADH1C/GSTP1/ADH1B |
| hsa04024 | cAMP signaling pathway | 4 | 8.13E-05 | CHRM1/DRD1/ADRB2/ADRB1 |
| hsa00980 | Metabolism of xenobiotics by cytochrome P450 | 3 | 8.87E-05 | ADH1C/GSTP1/ADH1B |
| hsa04725 | Cholinergic synapse | 3 | 0.000278 | CHRM1/ACHE/CHRM5 |
| hsa00350 | Tyrosine metabolism | 2 | 0.000821 | ADH1C/ADH1B |
| hsa00071 | Fatty acid degradation | 2 | 0.001227 | ADH1C/ADH1B |
| hsa04923 | Regulation of lipolysis in adipocytes | 2 | 0.002053 | ADRB2/ADRB1 |
| hsa00830 | Retinol metabolism | 2 | 0.002826 | ADH1C/ADH1B |
| hsa00010 | Glycolysis / Gluconeogenesis | 2 | 0.002909 | ADH1C/ADH1B |
| hsa04924 | Renin secretion | 2 | 0.002994 | ADRB2/ADRB1 |
| hsa04540 | Gap junction | 2 | 0.004827 | DRD1/ADRB1 |
| hsa04970 | Salivary secretion | 2 | 0.005154 | ADRB2/ADRB1 |
| hsa04261 | Adrenergic signaling in cardiomyocytes | 2 | 0.013374 | ADRB2/ADRB1 |
| hsa04022 | cGMP-PKG signaling pathway | 2 | 0.016622 | ADRB2/ADRB1 |
| hsa04810 | Regulation of actin cytoskeleton | 2 | 0.026292 | CHRM1/CHRM5 |

1. The detailed information of Cluster 5

| Pathway ID | Pathway name | Count | *P* value | Gene |
| --- | --- | --- | --- | --- |
| hsa03320 | PPAR signaling pathway | 3 | 4.56E-06 | RXRA/PPARD/RXRB |
| hsa04928 | Parathyroid hormone synthesis, secretion and action | 3 | 1.15E-05 | BCL2/RXRA/RXRB |
| hsa04920 | Adipocytokine signaling pathway | 2 | 0.000514 | RXRA/RXRB |
| hsa04659 | Th17 cell differentiation | 2 | 0.001235 | RXRA/RXRB |
| hsa04919 | Thyroid hormone signaling pathway | 2 | 0.001526 | RXRA/RXRB |

**Table S6: The GO and KEGG analysis on Triptoditerpenic acid B and DKD co-targets.**

1. GO enrichment analysis

| ID | Description | Count | *P* value | Gene |
| --- | --- | --- | --- | --- |
| GO:0009725 | response to hormone | 18 | 4.44E-15 | NOS2;CHRM3;CHRM1;ESR1;AR;CHRM5;PTGS2;CA2;RXRA;ADRA1A;PGR;CHRM2;ESR2;NR3C1;RXRB;CCNA2;NCOA2;NCOA1 |
| GO:0009719 | response to endogenous stimulus | 21 | 6.11E-15 | NOS2;CHRM3;CHRM1;ESR1;AR;CHRM5;PTGS2;CA2;RXRA;ADRA1A;PGR;CHRM2;ADRB2;OPRM1;ESR2;NR3C1;GSK3B;RXRB;CCNA2;NCOA2;NCOA1 |
| GO:0071495 | cellular response to endogenous stimulus | 19 | 8.56E-14 | CHRM3;CHRM1;ESR1;AR;CHRM5;PTGS2;CA2;RXRA;PGR;CHRM2;ADRB2;OPRM1;ESR2;NR3C1;GSK3B;RXRB;CCNA2;NCOA2;NCOA1 |
| GO:0007267 | cell-cell signaling | 20 | 8.82E-14 | NOS2;CHRM3;CHRM1;ESR1;AR;SCN5A;CHRM5;PTGS2;CA2;ACHE;ADRA1A;PGR;CHRM2;ADRA1B;ADRB2;ADRA1D;OPRM1;ESR2;DPP4;GSK3B |
| GO:0032870 | cellular response to hormone stimulus | 15 | 2.47E-13 | CHRM3;CHRM1;ESR1;AR;CHRM5;CA2;RXRA;PGR;CHRM2;ESR2;NR3C1;RXRB;CCNA2;NCOA2;NCOA1 |
| GO:0007188 | adenylate cyclase-modulating G protein-coupled receptor signaling pathway | 10 | 3.80E-12 | CHRM3;CHRM1;CHRM5;OPRD1;ADRA1A;CHRM2;ADRA1B;ADRB2;ADRA1D;OPRM1 |
| GO:0007187 | G protein-coupled receptor signaling pathway, coupled to cyclic nucleotide second messenger | 10 | 1.62E-11 | CHRM3;CHRM1;CHRM5;OPRD1;ADRA1A;CHRM2;ADRA1B;ADRB2;ADRA1D;OPRM1 |
| GO:0035690 | cellular response to drug | 11 | 1.69E-11 | NOS2;CHRM3;KCNH2;CHRM1;CHRM5;PTGS2;CHRM2;OPRM1;NR3C1;CCNA2;NCOA1 |
| GO:0048545 | response to steroid hormone | 11 | 5.28E-11 | ESR1;AR;PTGS2;CA2;RXRA;PGR;ESR2;NR3C1;RXRB;NCOA2;NCOA1 |
| GO:0033993 | response to lipid | 14 | 1.77E-10 | NOS2;ESR1;AR;PTGS2;CA2;RXRA;PGR;OPRM1;ESR2;NR3C1;RXRB;CCNA2;NCOA2;NCOA1 |

1. KEGG pathway analysis

| Pathway ID | Description | Count | *P* value | Gene |
| --- | --- | --- | --- | --- |
| hsa04080 | Neuroactive ligand-receptor interaction | 12 | 3.46E-10 | CHRM3;CHRM1;CHRM5;OPRD1;ADRA1A;CHRM2;ADRA1B;ADRB2;ADRA1D;OPRM1;NR3C1;PRSS1 |
| hsa04020 | Calcium signaling pathway | 9 | 3.02E-08 | NOS2;CHRM3;CHRM1;CHRM5;ADRA1A;CHRM2;ADRA1B;ADRB2;ADRA1D |
| hsa04915 | Estrogen signaling pathway | 7 | 1.03E-06 | ESR1;PGR;OPRM1;ESR2;HSP90AA1;NCOA2;NCOA1 |
| hsa04919 | Thyroid hormone signaling pathway | 6 | 6.29E-06 | ESR1;RXRA;GSK3B;RXRB;NCOA2;NCOA1 |
| hsa05200 | Pathways in cancer | 10 | 2.79E-05 | NOS2;ESR1;AR;PTGS2;RXRA;ESR2;GSK3B;HSP90AA1;RXRB;NCOA1 |
| hsa04970 | Salivary secretion | 5 | 2.90E-05 | CHRM3;ADRA1A;ADRA1B;ADRB2;ADRA1D |
| hsa04725 | Cholinergic synapse | 5 | 8.31E-05 | CHRM3;CHRM1;CHRM5;ACHE;CHRM2 |
| hsa04261 | Adrenergic signaling in cardiomyocytes | 5 | 2.72E-04 | SCN5A;ADRA1A;ADRA1B;ADRB2;ADRA1D |
| hsa05224 | Breast cancer | 5 | 2.99E-04 | ESR1;PGR;ESR2;GSK3B;NCOA1 |
| hsa04022 | cGMP-PKG signaling pathway | 5 | 4.82E-04 | OPRD1;ADRA1A;ADRA1B;ADRB2;ADRA1D |

**Table S7:The GO and KEGG analysis based on compound-DKD PPI network using WebGestalt** ^a.b.^

a.The GO enrichment analysis based on compound-DKD PPI network

| module | geneSet | description | count | pValue | FDR | gene |
| --- | --- | --- | --- | --- | --- | --- |
| cluster1 | GO:0033993 | response to lipid | 11 | 1.17E-11 | 1.07E-07 | NR3C1;PGR;ESR1;AR;JUN;MAPK14;PTGS2;MAPK8;VCAM1;NOS2;CD86 |
|  | GO:0034097 | response to cytokine | 11 | 1.05E-10 | 4.80E-07 | HSP90AA1;JUN;MAPK14;PTGS2;MAPK8;VEGFA;HMOX1;VCAM1;CXCR4;NOS2;CD86 |
|  | GO:0071345 | cellular response to cytokine stimulus | 10 | 1.53E-09 | 3.74E-06 | HSP90AA1;MAPK14;PTGS2;MAPK8;VEGFA;HMOX1;VCAM1;CXCR4;NOS2;CD86 |
|  | GO:0031328 | positive regulation of cellular biosynthetic process | 12 | 1.72E-09 | 3.74E-06 | NR3C1;PGR;ESR1;HSP90AA1;AR;JUN;MAPK14;PTGS2;VEGFA;HMOX1;NOS2;CD86 |
|  | GO:0009891 | positive regulation of biosynthetic process | 12 | 2.06E-09 | 3.74E-06 | NR3C1;PGR;ESR1;HSP90AA1;AR;JUN;MAPK14;PTGS2;VEGFA;HMOX1;NOS2;CD86 |
|  | GO:0009628 | response to abiotic stimulus | 10 | 4.09E-09 | 4.81E-06 | HSP90AA1;JUN;MAPK14;PTGS2;MAPK8;VEGFA;HMOX1;VCAM1;CXCR4;NOS2 |
|  | GO:1901700 | response to oxygen-containing compound | 11 | 4.36E-09 | 4.81E-06 | NR3C1;ESR1;AR;JUN;MAPK14;PTGS2;MAPK8;HMOX1;VCAM1;NOS2;CD86 |
|  | GO:0042127 | regulation of cell proliferation | 11 | 4.60E-09 | 4.81E-06 | PGR;ESR1;AR;JUN;MAPK14;PTGS2;VEGFA;HMOX1;VCAM1;NOS2;CD86 |
|  | GO:0032496 | response to lipopolysaccharide | 7 | 4.77E-09 | 4.81E-06 | JUN;MAPK14;PTGS2;MAPK8;VCAM1;NOS2;CD86 |
|  | GO:0002237 | response to molecule of bacterial origin | 7 | 6.30E-09 | 5.72E-06 | JUN;MAPK14;PTGS2;MAPK8;VCAM1;NOS2;CD86 |
| cluster2 | GO:0007188 | adenylate cyclase-modulating G protein-coupled receptor signaling pathway | 6 | 7.04E-09 | 3.14E-05 | OPRD1;CHRM2;ADRA1A;ADRA1D;ADRA1B;CHRM3 |
|  | GO:0042420 | dopamine catabolic process | 3 | 1.30E-08 | 3.14E-05 | MAOA;MAOB;SLC6A3 |
|  | GO:0007187 | G protein-coupled receptor signaling pathway, coupled to cyclic nucleotide second messenger | 6 | 1.69E-08 | 3.14E-05 | OPRD1;CHRM2;ADRA1A;ADRA1D;ADRA1B;CHRM3 |
|  | GO:0019614 | catechol-containing compound catabolic process | 3 | 2.07E-08 | 3.14E-05 | MAOA;MAOB;SLC6A3 |
|  | GO:0042424 | catecholamine catabolic process | 3 | 2.07E-08 | 3.14E-05 | MAOA;MAOB;SLC6A3 |
|  | GO:0006836 | neurotransmitter transport | 6 | 2.55E-08 | 3.32E-05 | MAOB;SLC6A4;CHRM2;SLC6A3;SLC6A2;ADRA1A |
|  | GO:0019336 | phenol-containing compound catabolic process | 3 | 4.44E-08 | 5.04E-05 | MAOA;MAOB;SLC6A3 |
|  | GO:0006940 | regulation of smooth muscle contraction | 4 | 9.23E-08 | 9.32E-05 | CHRM2;ADRA1A;ADRA1B;CHRM3 |
|  | GO:0042493 | response to drug | 8 | 1.53E-07 | 1.39E-04 | CYP3A4;MAOB;SLC6A4;CHRM2;SLC6A3;SLC6A2;ADRA1A;CHRM3 |
| cluster3 | GO:0033993 | response to lipid | 7 | 3.52E-07 | 0.003197 | ESR2;NCOA1;NCOA2;PPARG;AHR;CD80;SELE |
|  | GO:0010557 | positive regulation of macromolecule biosynthetic process | 8 | 2.49E-06 | 0.011327 | ESR2;NCOA1;NCOA2;PPARG;AHR;IL2;CD80;KDR |
|  | GO:0009891 | positive regulation of biosynthetic process | 8 | 4.11E-06 | 0.012458 | ESR2;NCOA1;NCOA2;PPARG;AHR;IL2;CD80;KDR |
|  | GO:0045893 | positive regulation of transcription, DNA-templated | 7 | 1.12E-05 | 0.021026 | ESR2;NCOA1;NCOA2;PPARG;AHR;IL2;CD80 |
|  | GO:0042493 | response to drug | 6 | 1.58E-05 | 0.021026 | NCOA1;NCOA2;PPARG;AHR;IL2;KDR |
|  | GO:1903508 | positive regulation of nucleic acid-templated transcription | 7 | 1.61E-05 | 0.021026 | ESR2;NCOA1;NCOA2;PPARG;AHR;IL2;CD80 |
|  | GO:1902680 | positive regulation of RNA biosynthetic process | 7 | 1.62E-05 | 0.021026 | ESR2;NCOA1;NCOA2;PPARG;AHR;IL2;CD80 |
|  | GO:0071396 | cellular response to lipid | 5 | 2.17E-05 | 0.02255 | ESR2;NCOA1;PPARG;AHR;CD80 |
|  | GO:0051254 | positive regulation of RNA metabolic process | 7 | 2.28E-05 | 0.02255 | ESR2;NCOA1;NCOA2;PPARG;AHR;IL2;CD80 |
|  | GO:0030522 | intracellular receptor signaling pathway | 4 | 2.48E-05 | 0.02255 | ESR2;NCOA1;PPARG;AHR |
| cluster4 | GO:0007188 | adenylate cyclase-modulating G protein-coupled receptor signaling pathway | 5 | 8.35E-08 | 7.59E-04 | CHRM5;ADRB1;DRD1;CHRM1;ADRB2 |
|  | GO:0007187 | G protein-coupled receptor signaling pathway, coupled to cyclic nucleotide second messenger | 5 | 1.73E-07 | 7.87E-04 | CHRM5;ADRB1;DRD1;CHRM1;ADRB2 |
|  | GO:0042311 | vasodilation | 3 | 6.26E-07 | 0.001576 | ADRB1;DRD1;ADRB2 |
|  | GO:0007190 | activation of adenylate cyclase activity | 3 | 6.93E-07 | 0.001576 | ADRB1;DRD1;ADRB2 |
|  | GO:0001659 | temperature homeostasis | 4 | 2.19E-06 | 0.003987 | ACHE;ADRB1;DRD1;ADRB2 |
|  | GO:0097755 | positive regulation of blood vessel diameter | 3 | 4.02E-06 | 0.006085 | ADRB1;DRD1;ADRB2 |
|  | GO:0007197 | adenylate cyclase-inhibiting G protein-coupled acetylcholine receptor signaling pathway | 2 | 6.80E-06 | 0.007933 | CHRM5;CHRM1 |
|  | GO:0071242 | cellular response to ammonium ion | 3 | 6.98E-06 | 0.007933 | CHRM5;DRD1;CHRM1 |
|  | GO:0001993 | regulation of systemic arterial blood pressure by norepinephrine-epinephrine | 2 | 9.06E-06 | 0.008235 | ADRB1;ADRB2 |
|  | GO:0002024 | diet induced thermogenesis | 2 | 9.06E-06 | 0.008235 | ADRB1;ADRB2 |
| cluster5 | GO:0048545 | response to steroid hormone | 4 | 2.89E-07 | 0.002632 | BCL2;PPARD;RXRA;RXRB |
|  | GO:0006367 | transcription initiation from RNA polymerase II promoter | 3 | 5.26E-06 | 0.010222 | PPARD;RXRA;RXRB |
|  | GO:0007565 | female pregnancy | 3 | 5.61E-06 | 0.010222 | BCL2;PPARD;RXRA |
|  | GO:0043401 | steroid hormone mediated signaling pathway | 3 | 5.70E-06 | 0.010222 | PPARD;RXRA;RXRB |
|  | GO:0033993 | response to lipid | 4 | 8.38E-06 | 0.010222 | BCL2;PPARD;RXRA;RXRB |
|  | GO:0014070 | response to organic cyclic compound | 4 | 8.64E-06 | 0.010222 | BCL2;PPARD;RXRA;RXRB |
|  | GO:0044706 | multi-multicellular organism process | 3 | 8.75E-06 | 0.010222 | BCL2;PPARD;RXRA |
|  | GO:0006352 | DNA-templated transcription, initiation | 3 | 1.04E-05 | 0.010222 | PPARD;RXRA;RXRB |
|  | GO:0009725 | response to hormone | 4 | 1.10E-05 | 0.010222 | BCL2;PPARD;RXRA;RXRB |
|  | GO:0009755 | hormone-mediated signaling pathway | 3 | 1.12E-05 | 0.010222 | PPARD;RXRA;RXRB |

b.The KEGG and Reactome pathway analysis based on compound-DKD PPI network

| module | geneSet | description | pValue | FDR | database | gene |
| --- | --- | --- | --- | --- | --- | --- |
| cluster1 | R-HSA-449147 | Signaling by Interleukins | 2.42E-11 | 4.97E-08 | pathway_Reactome | HSP90AA1;JUN;MAPK14;PTGS2;MAPK8;VEGFA;HMOX1;VCAM1;NOS2;CD86 |
|  | hsa05200 | Pathways in cancer | 8.41E-11 | 8.63E-08 | pathway_KEGG | ESR1;HSP90AA1;AR;JUN;PTGS2;MAPK8;VEGFA;HMOX1;CXCR4;NOS2 |
|  | hsa05418 | Fluid shear stress and atherosclerosis | 1.79E-10 | 1.22E-07 | pathway_KEGG | HSP90AA1;JUN;MAPK14;MAPK8;VEGFA;HMOX1;VCAM1 |
|  | R-HSA-1280215 | Cytokine Signaling in Immune system | 1.22E-09 | 6.27E-07 | pathway_Reactome | HSP90AA1;JUN;MAPK14;PTGS2;MAPK8;VEGFA;HMOX1;VCAM1;NOS2;CD86 |
|  | R-HSA-6785807 | Interleukin-4 and Interleukin-13 signaling | 2.74E-09 | 1.12E-06 | pathway_Reactome | HSP90AA1;PTGS2;VEGFA;HMOX1;VCAM1;NOS2 |
|  | R-HSA-2262752 | Cellular responses to stress | 1.66E-08 | 5.68E-06 | pathway_Reactome | NR3C1;PGR;HSP90AA1;AR;JUN;MAPK14;MAPK8;VEGFA |
|  | R-HSA-4090294 | SUMOylation of intracellular receptors | 4.97E-08 | 1.46E-05 | pathway_Reactome | NR3C1;PGR;ESR1;AR |
|  | R-HSA-8953897 | Cellular responses to external stimuli | 6.07E-08 | 1.56E-05 | pathway_Reactome | NR3C1;PGR;HSP90AA1;AR;JUN;MAPK14;MAPK8;VEGFA |
|  | hsa05167 | Kaposi sarcoma-associated herpesvirus infection | 7.19E-08 | 1.64E-05 | pathway_KEGG | JUN;MAPK14;PTGS2;MAPK8;VEGFA;CD86 |
|  | hsa04657 | IL-17 signaling pathway | 8.61E-08 | 1.77E-05 | pathway_KEGG | HSP90AA1;JUN;MAPK14;PTGS2;MAPK8 |
| cluster2 | R-HSA-375280 | Amine ligand-binding receptors | 6.30E-10 | 1.29E-06 | pathway_Reactome | CHRM2;ADRA1A;ADRA1D;ADRA1B;CHRM3 |
|  | R-HSA-373076 | Class A/1 (Rhodopsin-like receptors) | 1.92E-08 | 1.97E-05 | pathway_Reactome | OPRD1;C3;CHRM2;ADRA1A;ADRA1D;ADRA1B;CHRM3 |
|  | R-HSA-390696 | Adrenoceptors | 9.36E-08 | 6.41E-05 | pathway_Reactome | ADRA1A;ADRA1D;ADRA1B |
|  | R-HSA-112311 | Neurotransmitter clearance | 1.34E-07 | 6.86E-05 | pathway_Reactome | MAOA;SLC6A4;SLC6A3 |
|  | R-HSA-500792 | GPCR ligand binding | 2.04E-07 | 8.38E-05 | pathway_Reactome | OPRD1;C3;CHRM2;ADRA1A;ADRA1D;ADRA1B;CHRM3 |
|  | hsa04080 | Neuroactive ligand-receptor interaction | 2.70E-07 | 9.26E-05 | pathway_KEGG | OPRD1;CHRM2;ADRA1A;ADRA1D;ADRA1B;CHRM3 |
|  | hsa04020 | Calcium signaling pathway | 1.10E-06 | 3.24E-04 | pathway_KEGG | CHRM2;ADRA1A;ADRA1D;ADRA1B;CHRM3 |
|  | hsa04970 | Salivary secretion | 2.35E-06 | 6.03E-04 | pathway_KEGG | ADRA1A;ADRA1D;ADRA1B;CHRM3 |
|  | R-HSA-390648 | Muscarinic acetylcholine receptors | 1.17E-05 | 0.00267 | pathway_Reactome | CHRM2;CHRM3 |
|  | hsa05030 | Cocaine addiction | 2.00E-05 | 0.004106 | pathway_KEGG | MAOA;MAOB;SLC6A3 |
| cluster3 | R-HSA-211976 | Endogenous sterols | 1.86E-06 | 0.0023 | pathway_Reactome | NCOA1;NCOA2;AHR |
|  | R-HSA-1989781 | PPARA activates gene expression | 3.14E-06 | 0.0023 | pathway_Reactome | NCOA1;NCOA2;PPARG;AHR |
|  | R-HSA-400206 | Regulation of lipid metabolism by Peroxisome proliferator-activated receptor alpha (PPARalpha) | 3.36E-06 | 0.0023 | pathway_Reactome | NCOA1;NCOA2;PPARG;AHR |
|  | R-HSA-211897 | Cytochrome P450 - arranged by substrate type | 2.86E-05 | 0.014667 | pathway_Reactome | NCOA1;NCOA2;AHR |
|  | R-HSA-381340 | Transcriptional regulation of white adipocyte differentiation | 5.89E-05 | 0.023888 | pathway_Reactome | NCOA1;NCOA2;PPARG |
|  | hsa05200 | Pathways in cancer | 6.98E-05 | 0.023888 | pathway_KEGG | ESR2;NCOA1;PPARG;IL2;MMP1 |
|  | R-HSA-193807 | Synthesis of bile acids and bile salts via 27-hydroxycholesterol | 8.62E-05 | 0.025284 | pathway_Reactome | NCOA1;NCOA2 |
|  | R-HSA-159418 | Recycling of bile acids and salts | 9.85E-05 | 0.025284 | pathway_Reactome | NCOA1;NCOA2 |
|  | R-HSA-211945 | Phase I - Functionalization of compounds | 1.21E-04 | 0.025763 | pathway_Reactome | NCOA1;NCOA2;AHR |
|  | R-HSA-1368082 | RORA activates gene expression | 1.25E-04 | 0.025763 | pathway_Reactome | NCOA1;NCOA2 |
| cluster4 | R-HSA-375280 | Amine ligand-binding receptors | 1.24E-10 | 2.55E-07 | pathway_Reactome | CHRM5;ADRB1;DRD1;CHRM1;ADRB2 |
|  | hsa04020 | Calcium signaling pathway | 2.25E-07 | 2.31E-04 | pathway_KEGG | CHRM5;ADRB1;DRD1;CHRM1;ADRB2 |
|  | hsa04080 | Neuroactive ligand-receptor interaction | 1.76E-06 | 0.001204 | pathway_KEGG | CHRM5;ADRB1;DRD1;CHRM1;ADRB2 |
|  | R-HSA-373076 | Class A/1 (Rhodopsin-like receptors) | 3.80E-06 | 0.001953 | pathway_Reactome | CHRM5;ADRB1;DRD1;CHRM1;ADRB2 |
|  | R-HSA-390648 | Muscarinic acetylcholine receptors | 6.75E-06 | 0.002775 | pathway_Reactome | CHRM5;CHRM1 |
|  | hsa04024 | cAMP signaling pathway | 1.66E-05 | 0.005695 | pathway_KEGG | ADRB1;DRD1;CHRM1;ADRB2 |
|  | R-HSA-500792 | GPCR ligand binding | 2.04E-05 | 0.005985 | pathway_Reactome | CHRM5;ADRB1;DRD1;CHRM1;ADRB2 |
|  | R-HSA-390696 | Adrenoceptors | 2.43E-05 | 0.006187 | pathway_Reactome | ADRB1;ADRB2 |
|  | hsa00982 | Drug metabolism | 2.71E-05 | 0.006187 | pathway_KEGG | ADH1C;ADH1B;GSTP1 |
|  | hsa00980 | Metabolism of xenobiotics by cytochrome P450 | 3.19E-05 | 0.006552 | pathway_KEGG | ADH1C;ADH1B;GSTP1 |
| cluster5 | R-HSA-9006931 | Signaling by Nuclear Receptors | 8.59E-08 | 1.77E-04 | pathway_Reactome | BCL2;PPARD;RXRA;RXRB |
|  | R-HSA-5362517 | Signaling by Retinoic Acid | 1.92E-07 | 1.98E-04 | pathway_Reactome | PPARD;RXRA;RXRB |
|  | R-HSA-383280 | Nuclear Receptor transcription pathway | 3.06E-07 | 0.000209 | pathway_Reactome | PPARD;RXRA;RXRB |
|  | hsa03320 | PPAR signaling pathway | 1.01E-06 | 5.18E-04 | pathway_KEGG | PPARD;RXRA;RXRB |
|  | hsa05222 | Small cell lung cancer | 1.95E-06 | 8.02E-04 | pathway_KEGG | BCL2;RXRA;RXRB |
|  | hsa04928 | Parathyroid hormone synthesis, secretion and action | 3.00E-06 | 0.001026 | pathway_KEGG | BCL2;RXRA;RXRB |
|  | hsa05200 | Pathways in cancer | 4.21E-06 | 0.001236 | pathway_KEGG | BCL2;PPARD;RXRA;RXRB |
|  | R-HSA-200425 | Import of palmitoyl-CoA into the mitochondrial matrix | 8.19E-06 | 0.001871 | pathway_Reactome | PPARD;RXRA |
|  | hsa05226 | Gastric cancer | 8.20E-06 | 0.001871 | pathway_KEGG | BCL2;RXRA;RXRB |
|  | R-HSA-204174 | Regulation of pyruvate dehydrogenase (PDH) complex | 1.08E-05 | 0.002219 | pathway_Reactome | PPARD;RXRA |


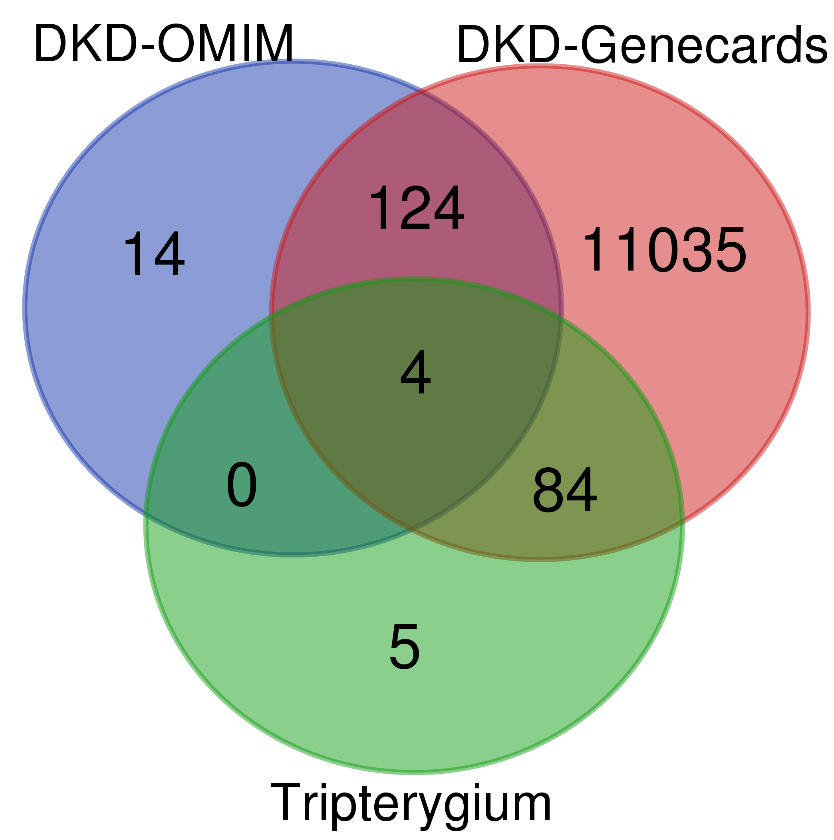


Figure S1: Matching of target genes between DKD and TwHF
